# IUGA International Guidelines on Obstetric Anal Sphincter Injuries

**DOI:** 10.1007/s00192-026-06642-3

**Published:** 2026-07-10

**Authors:** A. H. Sultan, N. A. Okeahialam, J. De Leeuw, L. Hickman, A. Madsen, G. Cundiff, L. Cattani, M. Giroux, S. Doumouchtsis, S. Lozo, H. Neels, G. Ganyaglo, M. Alarab, N. Rajmaheswari, C. Polle, R. Thakar

**Affiliations:** 1 Croydon University Hospital, London Road, Croydon, CR7 7YE UK; 2 City St George’s University of London, London, UK; 3 Brunel University of London, London, UK; 4 St Mary’s Hospital, Manchester, UK; 5 University of Liverpool, Liverpool, UK; 6 Wexner Medical Center, The Ohio State University, Ohio, USA; 7 University of Toronto, Toronto, Canada; 8 Mount Sinai Hospital, Toronto, Canada; 9 Chennai Urology & Robotic Institute, Chennai, India; 10 Korle Bu Teaching Hospital, Accra, Ghana; 11 Epsom and St Helier University Hospitals NHS Trust, London, United Kingdom; 12 National and Kapodistrian University of Athens, Athens, Greece; 13 American University of the Caribbean, Cupecoy, Sint Maarten; 14 Ross University, Mirarar, FL USA; 15 Antwerp University Hospital, Edegem, Belgium; 16 Columbia University Irving Medical Center, New York, USA; 17 Mayo Clinic, Rochester, MN USA; 18 Ikazia Hospital, Rotterdam, Netherlands; 19 University of British Columbia, Vancouver, Canada; 20 Department of Development and Regeneration, KU Leuven, Leuven, Belgium; 21 Zuyderland Medical Cente, Heerlen/Sittard-Geleen, The Netherlands; 22 Department of Obstetrics and Gynecology, Regina General Hospital, University of Saskatchewan, Regina, Canada; 23 Institute for Global and Reproductive Health, Nairobi, Kenya

## Summary

- Obstetric health care providers should have a basic understanding of the anatomy and physiology of anal continence in order to perform surgical repair, optimize nonsurgical management, and adequately counsel patients on the risks and outcomes of obstetric anal sphincter injuries (OASIs) (Grade Good Practice Point [GPP]).
- Patients should be fully informed of the potential for pelvic floor and anal sphincter injury with vaginal birth during the antenatal period, prior to experiencing labor. This counseling should include individual factors that can increase or reduce risks of injuries, including the risks and benefits of operative vaginal birth (OVB), episiotomy, and cesarean section (CS) (Grade GPP).
- Perineal protection strategies (e.g., perineal massage, warm compresses, perineal support at crowning) can be used during the second stage of labor to reduce OASI risk (Grades A–C).
- Clinicians should consider seeking support from a second clinician to assist with perineal trauma prevention techniques (Grade B).
- Mediolateral/lateral episiotomy during operative vaginal births in nulliparous women is associated with a lower risk of OASIs; the association in forceps-assisted births is stronger than in vacuum-assisted births (Grade A). Clinicians should ensure that the angle is 60° from the midline at crowning.
- Every woman should be offered a rectal examination after every vaginal birth to avoid missing OASIs or rectal buttonhole tears (Grade GPP).
- The Sultan classification should be used when describing any OASI to ensure that all injuries, especially internal sphincter injuries, are excluded (Grade A).
- Repair settings:
  1. Desirable: Repair should be conducted under general or regional anesthesia where there is access to good lighting, appropriate equipment and aseptic conditions (Grade GPP)
  2. Essential: Repair in the other settings may be performed under certain circumstances, provided that necessary requirements are made available, including adequate pain relief, access to good lighting, appropriate equipment, and aseptic conditions (Grade GPP).
- Repair of OASIs should be conducted by a doctor who has been formally trained or certified in primary anal sphincter repair (of all grades of third-degree and fourth-degree tears) or under the supervision of a certified trainer or specialist (Grade GPP).
- If there is no doctor of suitable expertise available to repair the OASI immediately, a delay can be considered, provided that there is no significant bleeding (Grade A).
- Women should be advised that up to 24 months following primary OASI repair, 82% are asymptomatic (Grade B).
- Women should be informed that the likelihood of experiencing anal incontinence increases with higher grades of OASI and with time following primary repair (Grade B).
- Clinicians should be aware that women are at risk of wound complications, including infection and dehiscence, following primary repair of OASIs (Grade GPP).
- If a woman is experiencing wound dehiscence of the primary OASI repair, referral to a specialist gynecologist or colorectal surgeon should be made for investigations, including endoanal ultrasound (EAUS) and consideration of conservative or surgical management (Grade GPP).
- Early secondary repair of a dehisced primary OASI repair can be offered to women. Women should be informed that there is a risk of wound dehiscence, wound infection, and fistula formation following early secondary repair of dehisced OASIs (Grade C).
- Both EAUS and anorectal manometry (ARM) can be used to help to differentiate laceration sequelae and inform discussion on future birth (Grade C).
  1. Advanced: Clinicians should be aware that there are published protocols to guide the mode of birth counseling in a subsequent pregnancy, which include symptom assessment, EAUS, and ARM (Grade B).
  2. Desirable: In the absence of EAUS and ARM, symptoms and a history of a fourth-degree tear can be used to guide the mode of birth counseling (Grade C).
- Elective CS may not be completely protective against the prevention of de novo or worsening short-term and long-term anorectal symptoms in women with prior OASI (Grade C).

## Section 1: Introduction

### Scope and objectives

The purpose of this guideline is to provide evidence-based guidance on the diagnosis, management, and treatment of obstetric anal sphincter injuries (OASIs). The guideline addresses prevention, identification, management, treatment of complications, and long-term follow-up of OASIs across various health care settings worldwide. It is intended for use by obstetricians, midwives, surgeons, and other health care providers involved in maternity care.

The primary objective of this guideline is to improve maternal health outcomes by providing evidence-based, applicable standards for the prevention, diagnosis, management, and follow-up of OASIs globally.

### Abbreviations

- **95% CI** 95% confidence interval
- **ACOG** American College of Obstetricians and Gynecologists
- **aOR** Adjusted odds ratio
- **ARM** Anorectal manometry
- **aRR** Adjusted relative risk
- **BMI** Body mass index
- **CS** Cesarean section
- **DRE** Digital rectal examination
- **DRESS** Digital Rectal Examination Scoring System
- **EAS** External anal sphincter
- **EAUS** Endoanal ultrasound
- **FGM-C** Female genital mutilation/cutting
- **GPP** Good Practice Point
- **IAS** Internal anal sphincter
- **IDC** Indwelling catheter
- **IUGA** International Urogynecological Association
- **LE** Lateral episiotomy
- **LMIC** Low- and middle-income countries
- **MD** Mean difference
- **MLE** Mediolateral episiotomy
- **MPP** Manual perineal protection
- **NNT** Number needed to treat
- **NSAIDs** Nonsteroidal anti-inflammatory drugs
- **OASI** Obstetric anal sphincter injury
- **OR** Odds ratio
- **OVB** Operative vaginal birth
- **PDS** Polydioxanone
- **POUR** Postoperative urinary retention
- **PUR** Postpartum urinary retention
- **PVR** Post-void residual
- **PVRBV** Post-void residual bladder volume
- **RCOG** Royal College of Obstetricians and Gynaecologists
- **RCT** Randomized controlled trials
- **rOASI** Recurrent obstetric anal sphincter injury
- **RR** Relative risk
- **SIG** Special interest group
- **SNM** Sacral neuromodulation
- **STPM** Superficial transverse perineal muscle
- **SVB** Spontaneous vaginal birth
- **SVD** Spontaneous vaginal delivery
- **TPUS** Transperineal ultrasound
- **VAS** Visual analogue scale
- **VBAC** Vaginal birth after cesarean section
- **WHO** World Health Organization

### Background and Epidemiology

#### Introduction

Obstetric anal sphincter injury (OASI) is a collective term that includes third- and fourth-degree perineal tears. It is a severe complication of vaginal birth involving injuries to the anal sphincter complex (third-degree tear) and, in some cases, the anal epithelium and rectal mucosa (fourth-degree tear). Recent increases in reported incidence have been documented but probably reflect improved training and detection [1]. OASIs are a leading cause of maternal morbidity, with potential long-term sequelae, including up to 50% long-term risk of anal incontinence, chronic perineal pain, sexual dysfunction, and psychological distress [2–4].

#### Pathophysiology and Clinical Impact

The anal sphincter complex comprises the internal anal sphincter (IAS), a smooth muscle maintaining resting anal tone, and the external anal sphincter (EAS), a skeletal muscle enabling voluntary control, together ensuring fecal continence. OASIs result from mechanical trauma during vaginal birth, often triggered by rapid fetal descent, excessive perineal stretching, or instrumental assistance [5]. The injury may involve direct tearing of muscle fibers, stretching beyond physiological limits, or compression of the pudendal nerve, leading to neuropathy.

Immediately after childbirth, women with OASIs may experience pain, wound infections and wound dehiscence [6]. Perineal pain and sexual difficulties, such as dyspareunia, are also common and affect recovery. Over time, OASIs can lead to more chronic issues. Fecal and flatal incontinence are more prevalent, as documented by long-term follow-up studies. Because symptoms may worsen with subsequent births, OASIs influence decisions about future childbirth modes [7, 8]. The above sequelae underscore the clinical significance of OASIs and the need for robust preventive and management strategies.

#### Epidemiology of Obstetric Anal Sphincter Injuries

The incidence rates of OASIs vary widely across populations, influenced by obstetric practices, diagnostic accuracy, maternal characteristics and demographics, data collection methodologies, and health care system factors. The prevalence of an OASI in the general population is between 0.25 and 6%. The prevalence of OASIs in primiparous women is between 1.4 and 16% and is higher than among the multiparous women (0.4 to 2.7%). In women with a history of previous OASIs, the risk of recurrence is higher and varies between 5.1 and 10.7% following subsequent childbirth [9].

Below, we summarize incidence rates from peer-reviewed epidemiological studies across different countries and regions.

##### United Kingdom

In England, a retrospective analysis of Hospital Episode Statistics (2000–2012) reported an overall increase in the reported incidence of OASIs from 1.8% to 5.9%, a trend attributed to improved detection rather than an actual increase in occurrence [1]. Similarly, a Welsh study using the Patient Episode Database for Wales (1999–2009) documented a rise from 1.81% to 5.62%, associated with increased use of episiotomy and forceps [10]. These findings suggest that heightened clinical awareness and shifts in birth practices might influence reported rates. The OASI Care Bundle, a quality improvement initiative implemented across 16 UK sites in 2017–2018, was aimed at reducing OASI rates through standardized perineal protection techniques and MLE where indicated. Gurol-Urganci et al. reported a pre-intervention incidence of 3.3% across 27,395 births, decreasing to 3.0% post-intervention (adjusted odds ratio [aOR] 0.80; 95% confidence interval [CI] 0.65–0.98) [11].

##### United States of America

In the USA, the reported incidence of OASIs is lower, possibly because of higher cesarean birth rates and inconsistent coding. A nested case–control study within a cohort of 5569 term vaginal births (2004–2008) at a tertiary center found a 4.5% incidence among primiparous women [12]. Another study reported an overall rate of 2.9%, with 5.8% among nulliparous and 0.6% among multiparous women [13]. In a retrospective cohort study of over 22,000 women undergoing term vaginal birth in Northern California, prolonged second-stage of labor, midline episiotomy, and vaginal birth after cesarean section (VBAC) were associated with an approximately threefold increased risk of OASIs [14]. Among primiparous or Asian women, the risk of OASIs was almost double.

##### Nordic Countries

Nordic countries provide robust, registry-based data. In Norway, a national prevention program aimed at reducing the incidence of OASIs was initiated in 2004 [15]. The implementation of this program resulted in a reduction in the incidence of OASIs from 4–5% to 1–2% [16]. A study from 2012 evaluated two time periods and confirmed a marked reduction from 4% to 1.9% following the implementation of perineal support [17]. In Sweden, a retrospective registry-based study found overall OASI rates of 3.5%. Primiparity, vacuum extraction, forceps, and high birthweight, increasing maternal age, decreasing maternal height, and previous CS were associated with increased risk of OASIs [18]. In Denmark, the incidence of OASIs in primiparous women increased from 6.1% in 2000 to 7.4% in 2010, whereas the episiotomy rate decreased and the use of vacuum extraction remained stable. Although the incidence of factors such as episiotomy and epidural varies significantly, their impact on the risk of OASIs appears stable over time [19]. However, another cohort study found that the incidence of OASIs decreased during the study period from 4.3% in 2011 to 2.6% in 2015, although there was no significant difference in the risk reduction between departments with and without formal prevention programs [20]. In Finland, the OASI rate has been below the Nordic average, but the rate has been on a steady rise, reaching an average of 1.0% in 2010, with a great variation between the birth units [21].

##### Australia

A population-based study in New South Wales found that the incidence of OASIs increased from 2.2% in 2001 to 2.9% in 2009 [1]. High birthweight, vacuum birth and Asian country of birth posed risks for all women. The greatest risk factor for an increase in trends was in women who had an Asian country of birth and vacuum-assisted birth, whereas the greatest trend amongst protective factors was an increase in maternal age ≥35 years for primiparas [22, 23]. 

##### Hong Kong

A retrospective study in Hong Kong found a prevalence of OASIs detected by endoanal ultrasound (EAUS) as high as 7.8% in the normal vaginal birth group and 5.6% in the instrumental birth group. However, this rather paradoxical difference was not statistically significant. The majority of operative vaginal births were vacuum-assisted births; hence, the authors interpreted these rates as similar in the two groups. Overall, 82.9% of women with OASIs on EAUS did not show clinical signs of OASIs, which were not diagnosed during clinical assessment immediately after birth. Birth weight was significantly higher in the OASI group. At 6–12 months after birth, 5.5% of women reported fecal incontinence and 17.9% reported flatal incontinence, but OASI was not associated with these symptoms [24].

##### Israel

Data from Israel report an exceptionally low incidence of 0.1–0.6% across vaginal births (2000–2016), a 10-fold reduction compared with Western countries [25]. This may reflect conservative obstetric practices (“extinct” forceps-assisted births and avoidance of midline episiotomies) or under-diagnosis as well as manual perineal protection (MPP), although structured training increased detection in some centers.

##### Netherlands

A recent population-based study from the Netherlands showed that use of mediolateral episiotomy (MLE) is associated with a reduction of OASIs in spontaneous vaginal births. Data from the Netherlands Perinatal Registry, involving 541,055 women, were analyzed and showed that the rate of OASI was 4.2% in 215,241 nulliparous and 1.4% in 325,814 multiparous women. In nulliparous and multiparous women, MLE was associated with a reduction of OASIs (aOR 0.3; 95% CI 0.30–0.34 and aOR 0.32; 95% CI 0.30–0.34 respectively). The association of MLE with a reduced rate of OASIs was stronger in high birthweight and in prolonged second stage groups. It was therefore proposed, based on these findings, that in nulliparous women, a MLE should be considered with an anticipated birth weight > 4000 g and a duration of the second stage of more than 60 min [26].

A previous study based on the Perinatal Registry found that incidences of OASIs following vacuum delivery in 130,157 primiparous women were 2.5% and 14% in those with and without a MLE respectively (aOR 0.14; 95% CI 0.13–0.15), and in 29,183 multiparous women these were 2.0% and 7.5% respectively (aOR 0.23; 95% CI 0.21–0.27) and concluded that MLE during both vacuum-assisted delivery and forceps delivery is associated with a fivefold to tenfold reduction in the rate of OASIs in primiparous and multiparous women respectively [23].

##### Austria

A nationwide study using the Austrian birth registry (2008–2014) reported an OASI incidence of 2.6% among primiparous women, rising from 2.1% in 2008 to 3.1% in 2014 [5]. This increase coincided with a decline in episiotomy rates (from 35.9% to 26.4%), underscoring the complex interplay between obstetric interventions and OASI risk [5].

##### Canada

**Fig. 1:**
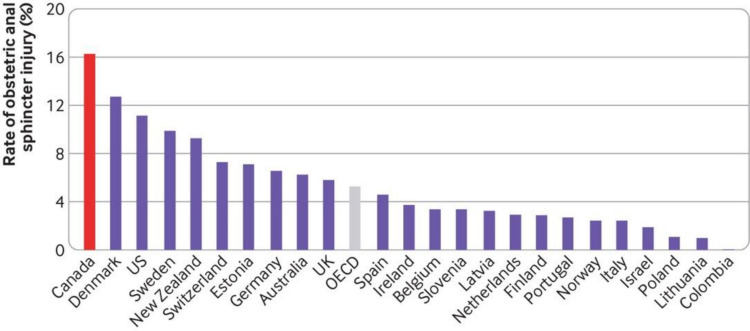
Obstetric anal sphincter injury (OASI) rates following operative vaginal births in 24 countries. One in 4 (25.3%) attempted forceps-assisted births and 1 in 8 (13.2%) attempted vacuum-assisted births appear to result in maternal (obstetric) trauma, most commonly OASI. *OECD* Organisation for Economic Co-operation and Development. Reproduced from Muraca et al. [28]

In Canada, a population-based study (2004–2015) showed an increasing trend from 4.2% to 5.2% [27]. An analysis by Muraca et al. [28] showed higher OASI rates following operative vaginal births (OVBs) than in many other countries (Fig. 1).

##### Low- and Middle-Income Countries

Data from low- to middle-income countries (LMICs) are limited. A systematic review that evaluated the rates of perineal trauma in low- and middle-income countries found a paucity of data for most countries. The Philippines has the highest reported OASI rate (15%), whereas Cambodia has the lowest (0.1%) [29]. A case–control study within a retrospective cohort of vaginal births at a tertiary care facility in South India (2008–2012) reported an OASI incidence of 2.1% after vaginal births. Major-degree (grade 3c- and fourth-degree) anal sphincter injuries constituted 20.9% of tears [30]. After adjusted analysis, significant predictors included primiparity, birth at or beyond 41 weeks of gestation, epidural analgesia, OVB, shoulder dystocia, birth weight ≥4000 g, and head circumference ≥35 cm. Episiotomy protected against OASIs, particularly in OVBs. Shoulder dystocia was significantly associated with major-degree tears, whereas episiotomy appeared to be protective. Retrospective cohort studies demonstrate that in Brazil, OASI rates range between 0.9% and 2.7% [31–34]. However, it is important to take into account that all prevalence data were collected prior to training in the detection of OASIs, meaning that these rates may be incorrect owing to underdiagnosis.

#### Trends and Temporal Changes

Globally, the reported incidence of OASIs has increased over recent decades in many countries. This rise is multifactorial, involving improved detection (e.g., through training and ultrasound), declining episiotomy rates, and population shifts (e.g., older maternal age, higher birth weights). However, real incidence may be underestimated in regions with less rigorous postpartum assessment, as missed injuries detected by EAUS can double reported rates (8–10%). [13, 24]

#### Challenges with Epidemiological Data

Current literature findings suggest that interpreting OASI incidence faces several obstacles:

- Diagnostic variability: Clinical under-detection is common, with several injuries missed without systematic rectal examination or imaging.
- Coding inconsistencies: Variations in International Classification of Diseases 10 coding skew national estimates.
- Study design: Retrospective data underestimate missed injuries, whereas prospective studies with ultrasound report higher rates.
- Intervention bias: Preventive strategies (e.g., perineal support, episiotomy use) alter incidence, confounding comparisons.

#### Conclusion

Obstetric anal sphincter injuries remain a critical obstetric challenge, with variations in incidence rates globally. This variability reflects the intersection among clinical, demographic, and systemic factors in its causation, suggesting that prevention of OASIs needs a multipronged approach.

### Anal Sphincter Anatomy and Physiology of Anal Continence

**Obstetric health care providers should have a basic understanding of the anatomy and physiology of anal continence in order to perform surgical repair, optimize nonsurgical management, and adequately counsel patients on the risks and outcomes of OASIs (GPP)**

Defecation and anal continence are achieved through the coordination of complex interactions between muscular, neural, hormonal, and cognitive factors that remain an area of ongoing research.

The convergence of teniae coli of the colon into a single longitudinal muscle marks the beginning of the rectum within the pelvis [35, 36]. The rectum ends at the anal canal, which is the distal-most 3 cm of the gastrointestinal tract. At this junction between the rectum and the anus, the puborectalis component of the levator ani muscles wraps posteriorly from the pubic symphysis like a sling to create an angle between the rectum and the anus (anorectal angle) [35, 37]. Continence is maintained, in part, by both the ability of the rectum to expand to hold stool and the maintenance of the anorectal angle. The distance between the pubic symphysis and puborectalis posterior to the anorectal angle seen on imaging is referred to as the levator hiatus and is an area of ongoing research in the pathophysiology of incontinence and prolapse [37].

The rectum is composed of inner circular and outer longitudinal smooth muscle layers. As they approach the anus, these layers converge with the IAS and conjoined longitudinal muscles respectively. Within the anal canal, the conjoined longitudinal muscle, vascular columns, and hemorrhoidal plexus maintain approximately 20% of the resting pressure of the anus [35, 36]. The remaining 80% of the resting pressure is maintained by the IAS, which forms the innermost circular layer of muscle around the anus [35, 38]. The actions of these structures are under autonomic innervation. In contrast, the EAS comprises skeletal muscle fibers with somatic innervation. Although its contribution to resting pressure is minimal, the EAS is responsible for controlling continence through active squeeze pressure.

The EAS has deep, superficial, and subcutaneous components with unique appearance on imaging studies [35–37, 39]. The deep EAS is continuous with the levator ani muscles and has a “U” shape on axial imaging. The superficial EAS encircles the anal canal and is the component most commonly identified for repair at the time of obstetric injury. The subcutaneous EAS inserts distally into the skin surrounding the anus and is responsible for the typical puckered appearance of the anal orifice. The IAS, EAS, and levator ani muscles, in addition to the perineal membrane, rectovaginal fascia, and perineal muscles, form the perineal body. The role of the perineal body in the pathophysiology of incontinence and prolapse is an area of ongoing research [39].

There are four main phases of defecation that are involved in an intact continence mechanism [35]:

1. The basal phase is the continent phase, which relies on an intact resting anal tone (primarily from the IAS), a preserved anorectal angle from the levator ani muscles (primarily the puborectalis), and an empty rectum.
2. In the pre-expulsive phase, the rectum fills and is able to expand with low pressures to sample the contents for solid, liquid, or gas, whereas the anus maintains its high-pressure tone through the rectoanal inhibitory reflex.
3. The expulsive phase is a reversal of these actions, with relaxation of the sphincters to dilate the anus and reduce anal pressure, relaxation of the levator ani to reduce the anorectal angle, and rectal contractions with or without Valsalva efforts to increase rectal pressure and facilitate evacuation.
4. The end phase is where a closing reflex results in a return to the basal resting state.

Many factors can influence this complex system of defecation and continence, including stool regularity and consistency, hormonal fluctuations, colonic dysbiosis, and other conditions affecting colonic motility [35, 36]. It is important to recognize that OASI not only disrupts the anatomy but also impacts this complex neuromuscular physiology. Surgical repair of the anatomy may not result in complete restoration of physiological function. Thus, primary prevention of OASIs is paramount.

### Scope of the Literature Search

The Cochrane Library was searched for relevant randomized controlled trials (RCTs), systematic reviews, and meta-analyses. MEDLINE and EMBASE were also searched from database inception to 2024, and the date of the last search was September 2024. The National Guideline Clearinghouse, ECRI Guidelines Trust, National Institute for Health and Care Excellence Evidence Search, Health Improvement Scotland, the Trip database, and the Guidelines International Network were searched for relevant guidelines and reviews.

The databases were searched using the relevant Medical Subject Headings, including all subheadings, and this was combined with a keyword search that included the terms: ‘human,’ ‘female,’ ‘childbirth,’ ‘obstetric,’ ‘perineum,’ ‘third degree,’ ‘fourth degree,’ ‘anal sphincter,’ ‘tear,’ ‘injury,’ ‘rupture,’ ‘damage,’ ‘incontinence,’ ‘faecal,’ ‘anal,’ ‘repair,’ ‘surgery,’ ‘sutures,’ ‘obstetric anal sphincter injury,’ ‘rectal buttonhole tear,’ ‘wound infection,’ ‘wound dehiscence,’ ‘episiotomy,’ ‘physiotherapy,’ ‘anal ultrasound,’ and ‘anal manometry.’

### Methodology

This guideline was developed in accordance with the Appraisal of Guidelines for Research and Evaluation II instrument. The scope of the guideline was determined by members of the International Urogynecological Association (IUGA) Obstetric Pelvic Floor and Anal Sphincter Injuries Special Interest Group (SIG) chaired by Abdul Sultan.

A multidisciplinary writing group was selected from SIG members. Special emphasis was placed on global representation, with members from LMICs actively involved in both authorship and review processes. Draft sections were reviewed by the SIG Guideline Advisory Committee to ensure clinical relevance and scientific accuracy. The drafts were circulated to the entire SIG membership for consultation and review, and the revised version was sent to the entire IUGA membership for comments. The finalized version was ratified by the IUGA Board. The guideline was developed independently of any commercial funding or external influence.

Identified evidence was critically appraised using the Scottish Intercollegiate Guidelines Network grading system and categorized by level and strength (Section “Levels of Evidence and Grades of Recommendations”). Where possible, recommendations are based on the highest-level available evidence. In the absence of published evidence, these have been annotated “Best Practice Point”, based on the clinical experience of the SIG. Practical algorithms and flowcharts are provided to facilitate implementation where appropriate, and tools such as audit criteria and checklists are proposed to support monitoring.

Barriers and facilitators to implementation were considered, especially in resource-limited settings. The guideline includes context-specific guidance for LMICs to support uptake in diverse settings. Patients’ perspectives were incorporated through a review of available patient-reported outcomes and values in the literature, and recommendations were aligned with known patient preferences where applicable.

### Levels of Evidence and Grades of Recommendation

Classification of evidence levels:

**Table:** 

1++	High-quality meta-analyses, systematic reviews of randomized controlled trials, or randomized controlled trials with a very low risk of bias
1+	Well-conducted meta-analyses, systematic reviews of randomized controlled trials, or randomized controlled trials with a low risk of bias
1–	Meta-analyses, systematic reviews of randomized controlled trials or randomized controlled trials with a high risk of bias
2++	High-quality systematic reviews of case–control or cohort studies or high-quality case–control or cohort studies with a very low risk of confounding, bias, or chance, and a high probability that the relationship is causal
2+	Well-conducted case–control or cohort studies with a low risk of confounding, bias, or chance and a moderate probability that the relationship is causal
2–	Case–control or cohort studies with a high risk of confounding, bias, or chance, and a significant risk that the relationship is not causal
3	Non-analytical studies, e.g., case reports, case series
4	Expert opinion

Grades of recommendation:

**Table:** 

A: At least one meta-analysis, systematic review or randomized controlled trial rated as 1++, and directly applicable to the target population; or a systematic review of randomized controlled trials or a body of evidence consisting principally of studies rated as 1+, directly applicable to the target population and demonstrating overall consistency of results
B: A body of evidence including studies rated as 2++ directly applicable to the target population, and demonstrating overall consistency of results; or extrapolated evidence from studies rated as 1++ or 1+
C: A body of evidence including studies rated as 2+ directly applicable to the target population, and demonstrating overall consistency of results; or extrapolated evidence from studies rated as 2++
D: Evidence level 3 or 4; or extrapolated evidence from studies rated as 2+

## Section 2: Classification and Diagnosis

### **Principles in clinical diagnosis**

Every woman must be offered a rectal examination after vaginal birth, and especially prior to suturing, to avoid missing an OASI or an isolated rectal buttonhole tear (GPP).

Informed consent should be obtained for a vaginal and rectal examination. If, after explanation of the reason for the rectal examination and the consequences of missing a diagnosis of OASI and a rectal buttonhole tear, the woman declines a rectal examination, it should be recorded in the notes (GPP).

There must be good exposure of the perineal injury, and if this is not possible, then the woman should be placed in lithotomy. Good lighting is essential (GPP).

If the examination is restricted because of pain, adequate analgesia must be given prior to the examination (GPP).

In the absence of regional anesthesia, the perineum should be visualized, and the woman should be asked to squeeze the anal sphincter muscles. Any asymmetry, particularly of the perianal skin overlying the corrugator cutis ani should be noted, as this could indicate disruption of the subcutaneous EAS (GPP).

The Sultan classification should be used when describing any OASI to ensure that all injuries, especially internal sphincter injuries, are identified (A).

If uncertainty exists between a 3a and 3b tear, the tear should be classified as 3b. Whether the tear is full-length or partial-length, it should still be graded according to its thickness because the technique of repair would remain unchanged (GPP).

**Fig. 2:**
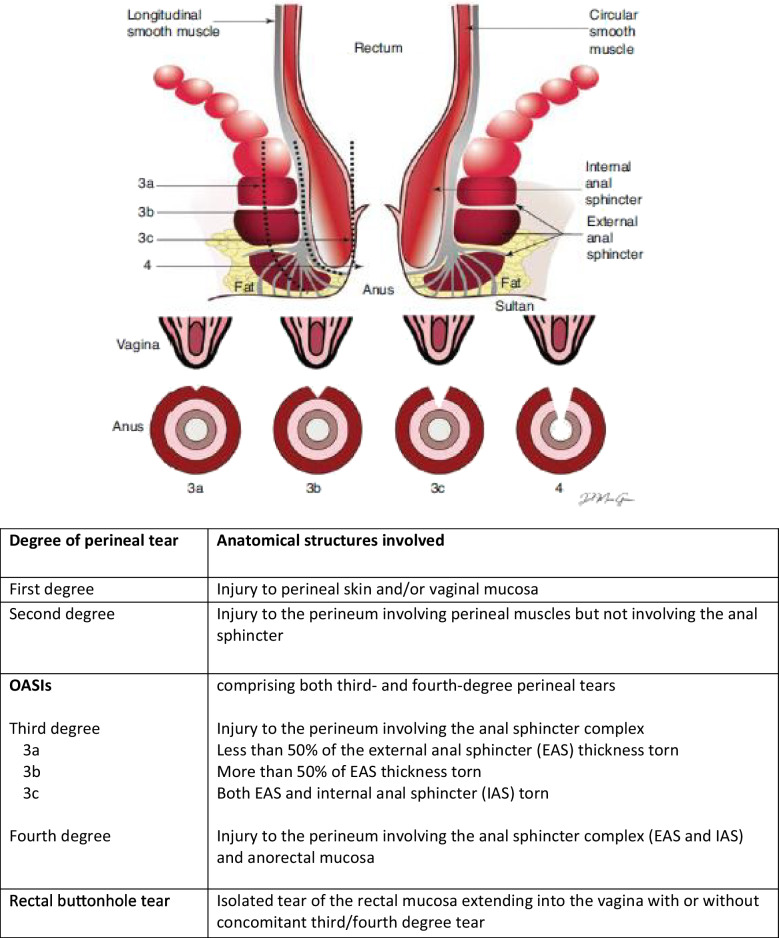
A schematic representation of the Sultan Classification of third and fourth degree tears. Note that the longitudinal muscle of the rectum continues as the conjoint longitudinal muscle. The lowermost images demonstrate the grade of tear according to the thickness of the injury to the external sphincter (brown) and any involvement of the internal sphincter (pink), irrespective of the length of the injury. Published with permission from Sultan and Thakar [48]

There has been variation in the classifications of perineal trauma in the past. A review conducted of obstetric textbooks at the Royal College of Obstetricians & Gynaecologists (RCOG) indicated that 17% did not include any classification system, whereas 22% categorized anal sphincter injuries as “second-degree” [40]. Sultan, therefore, introduced a standardized classification of perineal trauma [41] that has been adopted internationally [42, 43], including the RCOG [44] American College of Obstetricians and Gynecologists (ACOG) [45]. The Sultan classification quantifies the degree of damage according to the thickness of the external anal sphincter injury, as shown below and represented in Fig. 2 [46]. A network meta-analysis established the incidence of anal incontinence after OASIs according to the Sultan classification [47].

#### *EAS* external anal sphincter, *IAS* internal anal sphincter

The intact anal sphincter appears as a circular band of muscle that can be demonstrated by insertion of a finger in the anal canal. The mean length of the anterior anal sphincter in females is no more than 2.5–3 cm, corresponding to the length of the distal digit of the index finger [46]. However, it has been shown that doctors and midwives have poor accuracy in estimating the length of their index finger and the distal digit [49]. Compared with males, the anterior anal sphincter is shorter in females [50]. To identify OASIs and to avoid underdiagnosis [51] and overdiagnosis of OASIs [52] it is vital for doctors and midwives to be aware of the actual length and location of the anal sphincter. Sultan described a technique of pill-rolling [46] to identify the integrity of the anal sphincter during a rectal examination in acute perineal injuries, which involves rolling the anal sphincter between the index finger (up to the distal crease) in the anal canal and the thumb on the perineum or through the vaginal tear. An intact anal sphincter would flick off the thumb during pill rolling; This can be done with an intact perineum, but via a torn perineum. After identification of the sphincter ring, a full assessment would enable grading of the tear [39, 46]. When the external sphincter is completely disrupted, it can retract within its capsule. In this situation, the torn ends of the external sphincter need to be grasped and pulled up for repair [39, 46].

Prior to the advent of endoanal ultrasound (EAUS), clinically diagnosed OASIs had been reported in 0.6% of vaginal births [53]. However, original prospective studies using EAUS have shown that the majority of OASIs were being undiagnosed [46, 54], and this was attributed to confusion with classification [55, 56] but also to the lack of knowledge and training [56]. Using EAUS, it has subsequently been shown that with adequate training, nearly all of these injuries can be diagnosed clinically [57] (Evidence level 2+).

#### Under and over-diagnosed OASIs

Following the advent of endoanal ultrasound, Sultan et al. [54] demonstrated that 33% of women sustained OASIs that were not identified at birth. At that time, these were labelled as “occult” because it was assumed that these injuries seen on ultrasound could not be seen clinically. Older prospective studies [50] have identified “occult” injuries ranging between 20% and 41%. However, it has now been established that these injuries were, in fact, unrecognized at birth. Andrews et al. [57] reported a study in which 241 women having their first vaginal birth had their perineum re-examined by an experienced research fellow, and EAUS was performed immediately after birth, being repeated 7 weeks postpartum. The prevalence of clinically diagnosed OASIs increased from 11% to 25% (*n*=59). Every clinically diagnosed injury was identified by postpartum EAUS. At 7 weeks, no de novo defects were identified by ultrasound. This study concluded that most sphincter defects that have previously been designated as “occult” injuries were, in fact, injuries that were missed clinically at birth. It was alarming to find that 87% and 27% of OASIS were not identified by midwives and doctors respectively. Although it is likely that some of these would have been detected at the time of suturing of the tear, it was concerning that clinical recognition of OASIs was suboptimal (Evidence level 2+).

Roper et al. [51] studied 1056 women with OASIs and found that 11% of the injuries were under classified, and Sioutis et al. [52] found that 7% of women diagnosed with OASIs actually sustained second-degree tears; the superficial transverse perineal muscle (STPM) was probably mistaken for the EAS. A further reason for under-reporting by the accoucheur is the stigma associated with OASIs. In many units, OASIs constitute a risk-management trigger that may be regarded as punitive, and it is therefore a disincentive to accurate reporting (Evidence level 2+).

The introduction of hands-on workshops and audiovisual material [58, 59] has made a remarkable difference to the detection of OASIs, as Wong et al. have shown that the rate of clinical detection has doubled. However, the detection rate by midwives has not changed during this period, and therefore, a focused training program for all health care professionals, including midwives, is required [60].

### Physical examination, digital rectal examination technique

**Following a visual examination of the genitalia, the labia should be parted with the thumb and index finger of one hand, and a vaginal examination should be performed with the index and middle fingers of the other hand to establish the full extent of the vaginal tear. When multiple or deep tears are present, it is best to examine and repair in lithotomy. The apex of the vaginal laceration should always be identified (GPP).**

**A structured rectal examination should then be performed to exclude injury to the anorectal mucosa and anal sphincter. The vagina should be exposed by parting the labia with the thumb and index finger of the other hand. The whole index finger is then inserted into the rectum and elevated whilst moving from side to side, and this is continued down to the anal verge. Furthermore, a third- or fourth-degree tear may be present beneath what appears to be intact perineal skin, with a tear extending behind the fourchette, highlighting the need to perform a rectovaginal examination after every birth in order to exclude an OASI (GPP).**

**Spontaneous rupture of the anal sphincter is very unlikely to occur if there is no laceration involving the vagina and perineum, but this can only be excluded by performing a proper vaginal and rectal examination (GPP).**

External anal sphincter:

- Similar color to raw red meat.
- Has an upper border lying adjacent to the superficial transverse perineal muscle (STPM.)
- Measures 2.5–3cm in a woman.
- The subcutaneous component is traversed by the conjoint longitudinal muscle, giving rise to the corrugator cutis ani.
- Can be contracted voluntarily as it is innervated by the pudendal nerve.
- Damage can result in fecal urgency and urge incontinence.

Internal anal sphincter:

- Similar color to raw fish or raw chicken.
- Is a continuation of the circular muscle of the rectum and thickens to form the IAS, and therefore does not have a proximal border.
- Measures 3 cm in length and is separated from the EAS by the conjoint longitudinal muscle.
- The distal end of the IAS lies a few millimeters proximal to the distal end of the EAS.
- If the EAS is relaxed following regional or general anesthesia, the distal end of the IAS will appear to be at a lower level.
- It cannot be contracted voluntarily as it is under autonomic control.
- If the IAS or anal epithelium is torn, the EAS will invariably be torn, and therefore, a careful examination is mandatory.
- Damage gives rise to flatus incontinence and passive soiling.

The EAS is closely related to the STPM located at its proximal border. However, when the STPM is torn, the upper border of the EAS comes into clear view.

Care needs to be taken to avoid mistaking the STPM for the EAS. As can be seen, the STPM can be a substantial muscle and of a similar color. The only difference is the direction of attachment laterally toward the pubic rami [48, 61]. Failure to recognize this could result in either an overdiagnosis of an OASI or, if the STPM was repaired on the assumption that it was a disrupted EAS, an OASI could be missed, resulting in anal incontinence [62]. For this reason, Sultan described the “finger-lift test” such that a finger inserted in the anal canal should elevate as the ends of the torn EAS muscles are grasped and pulled anteriorly [63]. If the finger does not move upward, it is likely that the STPM, which is attached laterally, is being pulled (Evidence level 4).

## Section 3: OASI Repair and Short-Term Management

### Consent

**Patients should be fully informed of the potential for pelvic floor and anal sphincter injury with vaginal birth during the antenatal period, prior to experiencing labor. This counseling should include individual factors that can increase or reduce the risks of injuries, including the risks and benefits of OVB, episiotomy, and CS (GPP).**

**When an OASI is diagnosed on postpartum examination, the health care provider should explain the extent of the injury, the importance of the anal sphincter in maintaining anal continence, and the purpose of operative repair to improve outcomes (GPP).**

**Prior to operative repair for OASIs, all health care providers should obtain informed consent with written documentation of the conversation, which should include a description of the procedure, in addition to the purpose, risks, benefits, alternatives, and possible outcomes of repair (GPP).**

“Consent is a process that involves supplying the patient with enough information to make a fully informed decision” (Royal College of Obstetricians and Gynaecologists [RCOG] Consent Advice No. 6). In the ideal situation, time would allow for written consent to be obtained for all procedures, especially when the patient is to undergo general or regional anesthesia. If there is an emergency that prevents written consent, verbal consent is an acceptable alternative, but should be witnessed by another health care provider. When this occurs, the reasons for obtaining verbal instead of written consent should be documented. In the case of repairing a small perineal laceration at the bedside under local anesthesia following vaginal birth, verbal consent is often considered to be acceptable [64, 65].

Although proof of consent is achieved by signing a document, the essence of consent is arrived at through a process of information exchange and shared decision-making. In order for consent to be valid, the person must be fully informed of the nature and purpose of the proposed plan, voluntarily agree without coercion, and have the capacity to provide consent at that moment [66, 67]. It is up to the health care provider to assess if a patient has the capacity to provide consent. Although the patient may generally have the mental capacity, this can be altered temporarily in situations commonly encountered during labor or immediately postpartum, such as “panic, shock, fatigue, pain, or medication [66].” If a patient has agreed to a procedure while under distress and without time to fully consider the information, ask questions, and consider alternatives, this is an act of submission in a time of vulnerability [64–67]. Although it may be necessary in some situations, it is not equivalent to consent, which is the standard of care [64, 65].

Currently, the available literature suggests that there are gaps in every aspect of informed consent relevant to OASIs and pelvic floor dysfunction after vaginal birth. Patients lack baseline knowledge of the potential for pelvic floor injury and subsequent pelvic floor dysfunction after childbirth [68–71]. Education and counseling by health care providers in the antenatal period regarding mode of birth, OVB, episiotomy, and pelvic floor or anal sphincter injury is often inadequate or absent [68–74]. In many cases, consent is never obtained, and patients agree to the health care provider’s actions [73, 74]. Post-birth, patients may not be informed of the extent of their OASI or long-term sequelae [69, 70, 75]. Patients desire better access to information and autonomy regarding intrapartum events [70, 76, 77]. In one study on women’s experience of an OASI care bundle, many did not remember receiving the written antenatal education and reported a desire for more information. This indicates the importance of effective communication and the need to strive for more effective educational strategies [70]. Another study describing the patient experience during labor, cited ineffective communication, loss of autonomy, and lack of informed consent as the top forms of mistreatment leading to feelings of powerlessness and symptoms of anxiety, depression, and post-traumatic stress disorder [76]. These experiences and outcomes not only represent a gap in standard of care, but also expose health care providers to risk of litigation (See Section “Governance, Risk, and Litigation”).

Ideally, the consent process should begin in early pregnancy by eliciting a patient’s values and preferences and providing education as outlined in Section “Patient Education and Counseling.” Patients should be fully informed of the potential for pelvic floor and anal sphincter injury with vaginal birth prior to experiencing labor. They should also be aware of individual factors that can increase or reduce risks of injuries, including the risks and benefits of OVB, episiotomy, and CS. It is ideal for these situations to be discussed and decisions to be documented in the antenatal period and confirmed as needed during labor. Following childbirth, if a patient has sustained an OASI, they should be fully informed of the extent of their injury and should understand the risks, benefits, alternatives, and potential outcomes of surgical repair. The information provided in this document can be useful in implementing this informed consent process into obstetric practice.

A free resource to guide antenatal education is available through the RCOG as part of an evidence-based OASI care bundle (“Perineal tears and episiotomies in childbirth”) [11].

### Repair Techniques

#### General Principles

**Repair of OASIs should be conducted by a doctor who has been formally trained or certified in primary anal sphincter repair (of all grades of third-degree and fourth-degree tears) or under the supervision of a certified trainer or specialist (D).**

**Repair of the perineum should be undertaken as soon as possible to minimize the risk of bleeding and trauma-related inflammation of the perineum (GPP).**

**If no doctor of suitable expertise is available to repair the OASI immediately, a delay can be considered, provided that there is no significant bleeding (B).**

**Rectal examination should be performed after repair of all perineal tears, particularly to recognize inadvertent passage of sutures into the anorectal mucosa (unless there is a fourth-degree tear). If a suture is identified, it should be removed to minimize the risk of a rectovaginal fistula (GPP).**

**Detailed notes should be made of the findings and repair (GPP).**

The morbidity associated with OASI depends on the extent of injury, the technique and materials used for suturing, and the skill of the person performing the procedure. Therefore, it is important that practitioners are adequately trained to diagnose and repair perineal and OASIs adequately. The skills and knowledge of the operator are important factors in achieving a successful repair. Women should be referred to a more experienced health care professional if uncertainty exists as to the nature or extent of the trauma sustained. The randomized study by Nordenstam et al. [78] of 165 women randomized to immediate or delayed (8- to 12-h delay) demonstrated that there was no difference in anal incontinence at 12 months. Therefore, a delay in repair can be considered when there is no significant bleeding and a doctor with sufficient expertise is available. This is of particular importance in low-resource countries, where this would allow women to be transferred to a center with appropriate facilities and an experienced clinician [79] (Evidence level 1+).

The case–control study by Soerensen et al. [80] of the 22 women undergoing a delayed primary repair more than 72 h after delivery, compared with women with no OASI, demonstrated that even at this time period there were no immediate postoperative complications. However, the severity of incontinence was higher (Mean Wexner score 4.1 vs 1.1, *p* < 0.01), but there was no significant difference in quality of life. It is important to note that this cohort also included women undergoing an early secondary sphincter repair, up to 15 days following delivery. However, the authors found no difference in outcomes between delayed primary repair and early secondary sphincter repair (Evidence level 2+).

Completion of a pre-designed proforma and a pictorial representation of the tears prove very useful when notes are being reviewed following complications, audit, or litigation. A review of 19 legal cases regarding OASI complications in the USA found that a key factor aiding legal defense against medical negligence included diligent record-keeping [81].

#### Importance of Adequate Repair

**Clinicians should be aware that the persistence of sphincter defects following primary repair of OASI are directly associated with anal incontinence (B).**

An inadequate primary repair is directly associated with increased morbidity. Meta-analysis of 103 studies including 16,110 women found that following primary repair of OASIs, persistent sphincter defects were diagnosed on ultrasound in 55% (95% CI 45–63) and 38% (95% CI 33–43) experienced anal incontinence. Overall, women with a persistent anal sphincter defect had a three-fold risk of experiencing anal incontinence (odds ratio [OR]: 2.77 [95% CI 1.95−3.92]) [82] (Evidence level 2++).

#### Setting for Repair

**Desirable: Repair should be conducted under general or regional anesthesia where there is access to good lighting, appropriate equipment, and aseptic conditions (GPP).**

**Essential: Repair in other settings may be performed under certain circumstances, provided that necessary requirements are made available, including adequate pain relief, access to good lighting, appropriate equipment, and aseptic conditions (GPP).**

Repair in an operating theater will allow the repair to be performed under optimal conditions with appropriate instruments, adequate light, and an assistant. A general or regional (spinal, epidural, caudal) anesthetic provides analgesia and muscle relaxation, which are important pre-requisites to enable proper evaluation of the full extent of the injury. The inherent tone of the EAS can result in retraction of the torn muscle ends within its capsular sheath, and adequate muscle relaxation allows these to be grasped and retrieved, enabling a tension-free repair [63] (Evidence level 4).

In LMICs, births may be unattended or occur in community-based health care systems with no facilities for emergency obstetric services or infrastructures available to repair OASIs. Therefore, immediate repair in an operating theater may not be feasible. Under these circumstances, repair can be carried out in the delivery room with good analgesia, local/regional anesthetic, and an aseptic environment [79].

#### Which Suture Materials Should Be Used to Repair Obstetric Anal Sphincter Injuries?

**In the presence of a fourth-degree tear, the torn anal epithelium should be repaired with 3/0 polyglactin or poliglecaprone sutures (D).**

**The sphincter muscles (EAS IAS) should be repaired with 3/0 polydioxanone (PDS) dyed sutures, or if these are not available, modern braided sutures such as 2/0 polyglactin, with equivalent outcomes in terms of suture migration (GPP).**

**Surgical knots should be buried beneath the superficial perineal muscles to minimize the risk of knot and suture migration to the skin (B).**

Polydioxanone is a monofilament suture, with a maximum absorption time of 183–238 days, whereas polyglactin is a braided suture with a shorter maximum absorption time of 56–70 days. The tensile strength of the suture material is also important as it relates to the ability of the suture to resist tissue-induced stress and should balance the tensile properties of the tissue to improve healing. Tensile strength is determined by the number of filaments within the suture. As PDS is a monofilament, it loses its total tensile strength within 90 days. Polyglactin is a multifilament and loses its total tensile strength within 28 days [83]. Therefore, 3/0 polyglactin should be used to repair the anorectal mucosa as it may cause less irritation and discomfort than PDS sutures and has the tensile strength to resist tissue-induced stress from the passage of stool. Other monofilament suture materials, such as poliglecaprone (e.g., Monocryl™), can be considered as it loses its tensile strength within 21 days, although it has a maximum absorption time of 91–199 days. However, as it is a monofilament, the risk of bacterial colonization is reduced as the braids within the suture material have been shown to be a nidus for infection [84]. There is no place for the use of rapidly absorbing polyglactin (e.g., Vicryl Rapide) during repair of the anal sphincter as it has a maximum absorption time of 40–50 days and loses its total tensile strength within 14 days [83] (Evidence level 4).

A randomized controlled trial of 150 women comparing 3/0 polyglactin and 3/0 PDS for repair of the EAS found no differences in suture-related morbidity (need for suture removal owing to pain, migration, or dyspareunia) at 6 weeks or anal incontinence [85]. There are no randomized studies evaluating the optimal suture material for IAS repair. However, owing to the suture properties of PDS, including its delayed absorption time and being a monofilament, meaning less tissue trauma and infection, it is the most widely used suture (Evidence level 1−).

Suture migration and/or dyspareunia have been shown to occur in 20% of cases at 6 weeks following repair of the EAS with PDS [85]. Therefore, to minimize suture migration, care should be taken to cut suture ends short and ensure that the overlying superficial perineal muscles cover them. Women should be warned of the possibility of knot migration to the skin surface with long-acting and non-absorbable suture materials such as PDS. Symptoms that they may report include irritation/pain in the perineal body after primary repair. This can be managed in the outpatient setting under local anesthesia by trimming the exposed suture ends [63] (Evidence level 2++).

#### Which Techniques Should Be Used to Accomplish Repair of Obstetric Anal Sphincter Injuries?

**The full thickness of the torn anal epithelium should be repaired with a continuous nonlocking suture, with the knots secured on the vaginal aspect (D).**

**The IAS should be identified and, if torn, repaired separately from the EAS using an end-to-end repair with interrupted mattress sutures (C).**

**When the EAS is only partially torn (grade 3a and some grade 3b), then an end-to-end repair should be performed using two or three mattress sutures (D).**

**If there is a full-thickness, full-length EAS tear (some grade 3b, grade 3c, or fourth-degree), either an overlapping or an end-to-end method can be used with equivalent outcome (A).**

**Figure-of-eight sutures should be avoided during the repair of OASIs because they are hemostatic in nature and may cause tissue ischemia (GPP).**

**The perineal muscles should be reconstructed following repair of the anal sphincter to avoid a deficient perineum (D).**

Repair technique summary table:

**Table:** 

Site	Suture material	Technique
Anal epithelium	3/0 polyglactin	Continuous
Internal anal sphincter	3/0 polydioxanone or, if unavailable, 2/0 polyglactin	End-to-end
External anal sphincter partial thickness	3/0 polydioxanone or, if unavailable, 2/0 polyglactin	End-to-end
External anal sphincter full thickness and length	3/0 polydioxanone or, if unavailable, 2/0 polyglactin	End-to-end or overlap

##### Anal Epithelium

Anal epithelium: As the anal epithelium is less than 1 mm thick and adherent to the IAS, a robust repair is required to minimize the risk of suture disruption owing to distention during the passage of stool. This can be achieved using a continuous suture technique. The distal end of the repair should extend outside the anal verge so that it is easily accessible during repair of the perineal skin [86]. A subcuticular repair of the anal epithelium via the transvaginal approach has also been described, but as the anal epithelium is thin, this approach would not ensure a robust repair able to withstand distention during the passage of stool [63] (Evidence level 4).

##### Internal Anal Sphincter

 The presence of an IAS defect is predictive of anal incontinence. A prospective study of 500 women found that an IAS defect increased the risk of severe anal incontinence 5-fold (OR 5.1; 95% CI 1.5–22.9). Therefore, it is imperative that if injured, all attempts should be made to repair it [87]. The first description of IAS repair using an end-to-end technique was reported by Sultan et al. [88] in 1999. A subsequent retrospective study of 111 women in Norway demonstrated that separate repair of the IAS using an end-to-end technique resulted in 61.5% of women experiencing a significant improvement in continence at 3-year follow-up [89] (Evidence level 2+).

As the IAS has less fibrous tissue than the striated muscle of the EAS, it is more likely to tear when sutures are placed under tension with an overlap repair. Therefore, no attempt should be made to use an overlap repair for the IAS [88].

##### External Anal Sphincter

A Cochrane review including six trials (588 women) showed that there was no significant difference in flatal incontinence (relative risk [RR] 1.14; 95% CI 0.58–2.23), three trials, *n* = 256) and fecal incontinence (RR 0.37; 95% CI 0.03–4.68), three trials, *n* = 256) between an end-to-end and an overlap repair technique at 12 months (Evidence level 1++).

However, the overlap repair of the EAS compared with end-to-end repair appears to be associated with lower risks of developing fecal urgency (RR 0.12; 95% CI 0.02–0.86, one trial, 52 women), anal incontinence symptoms (standardized mean difference –0.70; 95% CI –1.26 to –0.14), and deterioration of anal incontinence symptoms (RR 0.26; 95% CI 0.09–0.79, one trial, 41 women). But, at the end of 36 months, there appears to be no difference in flatus (RR 1.12; 95% CI 0.63–1.99, one trial, 68 women) or fecal incontinence between the two techniques (RR 1.01; 95% CI 0.34–2.98, one trial, 68 women) There is also a suggestion that the overlap repair is more robust in the longer term. However, as there was considerable heterogeneity in selection criteria (some studies included grade 3a tears and partial thickness grade 3b tears), the outcome measures, time points, and reported results, more research evidence is needed in order to confirm or refute these findings [90]. Therefore, an end-to-end or overlap repair of full-thickness EAS tears may be performed at the discretion of the trained clinician (Evidence level 4).

An overlap repair allows for a greater surface area of contact between the muscle ends, meaning that if further retraction of the overlapped muscle ends were to occur, muscle continuity is likely to be maintained. With an end-to-end repair, as the full length of the EAS may not be identified, this may lead to incomplete apposition, a shorter anal canal length, and increased risk of developing fecal urgency and incontinence [63]. A prospective study of 74 women followed up an average of 27 months following primary repair found that the resulting anal sphincter length following repair was significantly associated with anal incontinence [91]. However, an overlap technique can only be performed when the full thickness of the EAS is involved and the full length of the EAS is free. This means that an overlap repair is not possible with a grade 3a tear or a partial thickness grade 3b tear; an end-to-end repair should be performed for all partial-thickness EAS tears (grade 3a and some grade 3b) [63]. As “figure-of-eight” sutures are hemostatic and can cause strangulation of tissue, they should never be used to repair the anorectal epithelium or the sphincters (Evidence level 2+).

After repair of the anal sphincter the perineal muscles should be reconstructed, as a short deficient perineum increases the risk of a repeat OASI in a subsequent vaginal birth [63] (Evidence level 4).

#### Which Techniques Should Be Used to Accomplish Repair of Isolated Rectal Buttonhole Tears?

**The full thickness of the torn anal epithelium should be repaired with a continuous nonlocking suture with the knots secured on the vaginal aspect (D).**

**The rectovaginal fascia should be approximated using an interrupted mattress or continuous technique with a 2/0 or 3/0 PDS suture to minimize the risk of a persistent recto-vaginal fistula (D).**

**The overlying vaginal skin can then be repaired with a continuous nonlocking 2/0 Vicryl suture (A).**

A rectal buttonhole tear is an isolated tear of the rectal mucosa and vagina [1, 2]. When an OASI involving the anal epithelium is in continuity with a rectal mucosal tear, it should be called a fourth-degree tear. However, when a third- or fourth-degree tear occurs concomitantly with an isolated rectal buttonhole tear, with an island of intact rectal mucosa between the third-/fourth-degree tear and the isolated rectal buttonhole tear, it should be classified and described as such: namely, rectal buttonhole tear plus the OASI grade [63, 92–94].

The true incidence of rectal buttonhole tears is unknown because of a failure to recognize these injuries, variations in classification (fourth-degree tear), and a reluctance to publish poor outcomes [93]. It is reported to occur in a wide range of vaginal births 1:1700 [5], 1:7000 [2], and 1:30,000 [93] vaginal births (Evidence level 3).

A review and case series of 12 women with isolated rectal buttonhole tears found that 1 case described a single-layer closure, 5 described a two-layer closure, and 6 described a three-layer closure. Two cases were repaired in collaboration with colorectal surgeons. Eleven women had an uncomplicated postoperative recovery between 6 weeks and 3 months. One patient had a defunctioning stoma at a later date owing to a second breakdown of the rectovaginal fistula repair [92]. The intervening rectovaginal fascia should be approximated using interrupted or continuous PDS 2/0 or 3/0 sutures to prevent the formation of a persistent rectovaginal fistula [63] (Evidence level 3).

Prior to repair of the rectal buttonhole tear, it is important to ensure that both the proximal and distal ends of the tear are clearly visualized. The repair should then be performed via the transvaginal approach in three layers (open access video available) [92]. The steps of repair are as follows. [63]. First, the mucosal tear should be sutured similarly to the anorectal mucosal repair of a fourth-degree tear with a full-thickness continuous Vicryl 3–0 suture. Second, the intervening rectovaginal fascia must be approximated using interrupted or continuous PDS 2–0 or 3–0 sutures. This is an essential step to minimize the risk of development of a rectovaginal fistula. Third, the vaginal skin should be closed using a continuous Vicryl or Vicryl Rapide 2–0 suture.

On rare occasions, an isolated rectal buttonhole tear can occur in association with a third-degree tear, in which case it should be classified as such, and the repair should be done separately for each. A colostomy is rarely indicated but should be considered following discussion with the colorectal surgeon in the presence of a large rectal tear (±7 cm) extending above the pelvic floor or if there is gross fecal contamination of the wound [63]. There is a risk of requiring a colostomy following a wound breakdown, but this is uncommon (occurring in less than 5–10% of cases). However, this information should be included during the counseling of a woman undergoing a rectal buttonhole tear repair [63].

### Perioperative Management

#### Preoperative Counseling

**Obtain informed consent in accordance with local guidelines and protocols. Where available, introduce all the disciplines to be involved in a patient’s management (GPP).**

**Patients should be encouraged to report any difficulties or concerns to the health care team, particularly in the postoperative period. Discuss prophylactic antibiotic use, postoperative bowel regimen, bladder catheterization, and provide assurances around pain management (GPP).**

**To minimize anxiety and ensure cooperation, involve the patient in the decision-making processes. Discuss postoperative follow-up and rehabilitation plans. Ensure adequate documentation of all discussions (GPP).**

#### Preoperative Assessment

**Be sure to document clinical examination findings pertinent to the diagnosis of OASI. Assess the patient’s overall health, namely, medical history and comorbidities (GPP).**

#### Intraoperative Management

**The use of intravenous broad-spectrum antibiotics is recommended during repair of OASIs to reduce the incidence of postoperative infections and wound dehiscence (B).**

The vagina and anal verge host a range of bacteria as normal flora. Raw surfaces, as occurs in OASI, can become seeded with bacteria, which then multiply. This can start a puerperal infection (localized or ascending). Infection will slow down healing post-OASI repair. The use of prophylactic antibiotics is aimed at reducing the risk of postoperative infections and potential wound breakdown, thus promoting wound healing and faster recovery. Prophylactic antibiotics should be administered within 1 h prior to repair to achieve adequate tissue concentrations at the time of repair. Regarding the choice of antibiotic, evidence-based local guidelines may apply, but common choices for prophylactic antibiotics include broad-spectrum intravenous agents that target skin flora, such as cephalosporins and penicillin derivatives (in cases of penicillin allergy, clindamycin or vancomycin may be considered) [95, 96] (Evidence level 2++).

#### Postoperative Management

**Postoperative laxatives are recommended to reduce the risk of wound dehiscence (C).**

**An indwelling catheter should be inserted and removed when the patient has recovered from anesthesia and is mobile (GPP).**

**Effective analgesia is critical to facilitate recovery, promote mobility, and improve the overall OASI repair experience (GPP).**

To prevent constipation and subsequent straining at bowel movement, which can disrupt the repair, there is agreement that stool softeners can be helpful. In the immediate postoperative period (first 24–48 h) mild laxatives, e.g., polyethylene glycol, lactulose (dose titrated to maintain soft, formed stools and not liquid stools), should be used. Locally available agents should be reviewed and considered for their suitability. Diet can also be modified with high-fiber diets (wholegrain), fruits, and vegetables. Encourage increased fluid intake and maintain adequate hydration. These tend to produce soft stools that will be easy to pass (Evidence level 2−).

Owing to perineal discomfort, pain, and the effect of regional anesthesia, the patient is unlikely to be able to void by herself [97, 98]. Continuous drainage of the bladder is the norm, but only until the patient has fully recovered from anesthesia and is ambulant. This may take 6–12 h post-repair, but longer if postoperative complications arise, e.g., hematoma. Adequate intraoperative hemostasis and postoperative analgesia should enable the patient to transition smoothly to self-voiding. Measuring voided volumes can be used to monitor urine output (Evidence level 1+).

For analgesia, treatment should be individualized based on needs and response. Multimodal approaches have proven more effective [99]. Acetaminophen and nonsteroidal anti-inflammatory drugs (NSAIDs), such as ibuprofen or ketorolac, are recommended first-line remedies. This may be oral or systemic, with the oral route preferred over the rectal. This minimizes the need, risks, and side effects associated with opioid use. Opioid medications may be considered if acetaminophen and NSAIDs prove ineffective. The lowest effective opioid dose over the minimum duration required should be utilized. However, it is important to note that opioids can cause constipation, which can lead to disruption of an anal sphincter repair. For women who labored with an epidural, labor analgesia, epidural morphine ≤2 mg, corresponding to the lowest effective dose, is recommended. Monitoring of the respiratory rate is, however, mandatory. Nonpharmacological approaches—cryotherapy (ice and chemical cold packs) and transcutaneous electrical nerve stimulation (adjuvants)—have been shown to be helpful [100]. Regular assessment of pain levels should be conducted using standardized pain scales and involve the patient in open discussions, allowing for timely adjustments to the pain management plan as needed (Evidence level 4).

#### Postpartum Bladder Protocol and Management of Voiding Dysfunction

**The risk of initial and persistent bladder overdistension can be reduced by implementing routines for observation for signs of bladder filling and emptying in the early postpartum period, as well as the use of bladder scans post-micturition in women with OASI to assess for successful bladder emptying (D).**

Postpartum urinary retention (PUR; also known as voiding dysfunction) is a common problem, defined as the inability to completely void after giving birth. If voiding dysfunction is not recognized, bladder overdistension can lead to denervation, detrusor atony, and prolonged voiding dysfunction.

In 2014, the results of a large retrospective analysis of postpartum women were published. Routinely collected post-void residual bladder volumes (PVRBVs) were reported using a bladder scanner at 4 and 6 h and 2–3 days post-birth or following removal of an indwelling catheter (IDC). A PUR incidence of 5.1% was found, with a residual volume measured of >150 ml. OASIs (aOR 2.01; 95% CI 1.09–3.72) were a significant independent predictor of PUR. The authors concluded that following the introduction of a protocol of timed voiding and routine measurement of PVRBV after birth/removal of the IDC, PUR is uncommon. Adopting a risk-factor-based approach to PVRBV screening was not supported by their data [101] (Evidence level 2+).

A more recent retrospective multicenter study confirms the above-mentioned need for further research. Tiberon et al. [102] found that in the absence of a consensus definition of PUR, the incidence varies substantially from author to author, ranging from 0.9 to 17.9%; in addition, they also studied persistent PUR (when it requires catheterization for more than 72 h), which concerns only 0.2% of births. They conclude that time in the management of urinary retention can cause bladder overdistension that can substantially delay its resolution, and suggest that more attentive monitoring of voiding could reduce the duration of this complication, thereby improving patient comfort and minimizing long-term complications (Evidence level 2+).

Ain et al. [103] found an overall prevalence of 12.9% of PUR after vaginal birth, and the prevalence of overt and covert PUR was 1.8% and 11.04% respectively, in a cohort of 878 women. Primigravidae, perineal injury, OVB, birth weight >3.5 kg, and a longer duration of the second stage of labor constituted risk factors for developing PUR. The authors also confirm that any misdiagnosis or delay in diagnosis of PUR can cause bladder overdistension, leading to irreversible detrusor damage. Hence, according to Ain et al., vigilant monitoring and early detection of PUR help in facilitating timely interventions and prevention of immediate and long-term sequelae (Evidence level 2−).

A Nigerian prospective longitudinal study in 250 women found an incidence of PUR of 17.6%. Women with PUR had a longer duration of the first stage (591 vs 501 min; *p* = 0.001) and the second stage (50 vs 32 min; *p* < 0.001) of labor than those without PUR. There was evidence that augmentation of labor (*p* < 0.01), catheterization during labor (*p* < 0.01), perineal injury (*p* < 0.01), and episiotomy (*p* < 0.01) were associated with the development of PUR. Also, women with PUR were more likely to experience storage and obstructive urinary symptoms than those without PUR. Resolution of PUR occurred within 24 h [104] (Evidence level 2+).

A large Chinese retrospective case–control study included primiparae who delivered vaginally between 1 July 2017 and 30 June 2019. Of the 12,609 women included in that study, 677 were diagnosed with overt PUR (incidence 5.37%). The authors drew similar conclusions: PUR was highly associated with epidural analgesia, forceps-assisted birth, vulvar oedema, episiotomy, and second-degree perineal tears (OR 3.42; 95% CI 2.37–4.94), a significant independent risk factor. They argue that more attention should be paid to women at a high risk to reduce the incidence of PUR. Unfortunately, no data were available about third- or fourth-degree tears [105] (Evidence level 2+).

Postoperative urinary retention (POUR) is the inability to void despite a full bladder in the postoperative period, often characterized by impaired bladder emptying with a rise in the volume of retained urine. Postoperative urinary retention could result from the effects of surgery or drugs used in the perioperative period. Effects of surgery, such as tissue edema, inflammation, denervation, and postoperative pain, alter the bladder physiology, leading to POUR. There are various ways of determining POUR, but the standard is measurement of post-void residual (PVR), either by in-and-out catheterization or by bladder ultrasound. Undiagnosed or untreated POUR can lead to significant morbidities such as discomfort, urinary tract infection, prolonged bladder distension with associated detrusor injury, and ureteric reflux resulting in renal dysfunction. It may even cause breakdown of the repair. POUR also causes the patient worry and anxiety [106].

A prospective cohort study of women who underwent delayed primary sphincter repair (≥ 3 months) was aimed at determining the incidence and risk factors for POUR following surgery for perineal tears, and to determine the time to normal voiding after POUR. Postoperative care was the same for all the participants. They all had an indwelling Foley catheter inserted at the end of the surgery. This catheter was either removed within 24 h after surgery (early catheter removal) or 24 h post-surgery (delayed catheter removal), depending on the attending surgeon. Antibiotics and non-opioid analgesia (paracetamol and NSAIDs), fluid administration, and ambulation were given to all the participants, which is a routine care protocol. An incidence of 19.6% was found, and the risk factors were repeat perineal-tear surgery and early urinary catheter removal after surgery. The median time to recovery of POUR (normal voiding) in this study was 42.4 (range 24–72) hours. The type of perineal-tear surgery may be a risk factor for POUR, with fourth-degree perineal tears that involve extensive dissection and a longer duration carrying a higher risk than second-degree perineal tears; however, insufficient data were collected to confirm this assumption. Nevertheless, the current evidence shows that POUR following surgery for perineal tears is common. The time to return to normal voiding in patients with POUR is short. Delayed urinary catheter removal could be considered, especially in patients undergoing repeat perineal-tear surgery. Future randomized controlled clinical trials among perineal-tear surgeries comparing rates of POUR between early and delayed catheter removal need to be conducted to confirm this guideline [106] (Evidence level 2++).

**Health care professionals should be aware that postpartum bladder problems and voiding dysfunction may influence a woman’s decision on mode of birth after previous OASI (C).**

Maternal requests for CS are increasing, partly because of a previous OASI. Narice et al. [107] aimed to understand which factors have the greatest influence in driving women’s decision-making process once they are pregnant after having sustained an OASI. They found that pregnant women with ongoing urinary symptoms rather than sexual or bowel dysfunction seem to favor a planned CS, regardless of the severity of their previous tear. Most women in their descriptive quantitative and qualitative research had experienced grade 3a/3b OASIs (*n* = 49, 77.78%), and they were more likely to report bladder pain and difficulties with voiding than those with major tears (*p* < 0.05). The type of tear did not directly impact the preferred mode of birth. However, women still experiencing bladder pain were more likely to request a CS (*p* < 0.05). Therefore, postpartum follow-up of urinary symptoms and bladder pain in a perineal trauma clinic is extremely valuable (Evidence level 2+).

#### Pelvic Floor Physiotherapy

##### Antenatal

**Women should be advised that evidence supporting antenatal physiotherapy in reducing the rate of OASI is conflicting (B).**

Evidence for the preventive effect of pelvic floor muscle exercises on OASI is controversial.

In a single-blind quasi-randomized controlled trial including 466 women, standard care alone, in comparison with daily perineal massage and pelvic floor exercises from 32 weeks of pregnancy until birth, was associated with significantly lower rates of episiotomies (OR 0.20; 95% CI 0.14–0.36) when compared with women who received standard of care only. In addition, this study demonstrated a reduction in third- (5.18% vs 13.12%, *p *< 0.001) and fourth-degree tears (0.52% vs 2.5%, *p* < 0.001) [108] (Evidence level 1+).

A systematic review and meta-analysis from 2020, including 16 studies (*n* = 2829 women), concluded that antenatal pelvic floor muscle exercises may be effective in reducing OASIs (RR 0.57; 95% CI 0.38–0.84). But these findings need to be interpreted taking the risk of bias of the studies included (moderate to high risk) into consideration. More high-quality RCTs are needed [109] (Evidence level 1−).

Another systematic review and meta-analysis, published in 2021 and including 9 studies (*n* = 1866 women), was aimed at evaluating whether pelvic floor muscle training prevents perineal laceration and concluded that pelvic floor muscle training has no effect on perineal trauma. No subgroup analysis was performed for OASIs. Further high-quality studies with a low risk of bias are needed to investigate different protocols and interventions [110] (Evidence level 1−).

A recent systematic review and meta-analysis of randomized clinical trials, including 30 studies (*n* = 6691 women), also studied the influence of pelvic floor muscle training alone or as part of a general physical activity program on OASIs. They concluded that pelvic floor muscle training during pregnancy proves to be an effective preventive intervention for reducing the risk of the occurrence of OASIs (RR 0.50; 95% CI 0.31–0.80). These authors highlighted the importance of incorporating pelvic floor muscle training into antenatal care and training programs to improve maternal well-being and overall childbirth outcomes. However, there was an unclear/high risk of bias in 24 studies [111] (Evidence level 1−).

##### Postpartum

**Women should be advised that physiotherapy following repair of OASIs could be beneficial (B).**

**As there are considerable variations in the availability of physiotherapy in different hospitals, the follow-up regimen is best designed according to local facilities (GPP).**

There was one RCT including 120 women comparing early home biofeedback therapy and pelvic floor exercises for women who sustained OASIs, and this study showed that there was no added value in using early home biofeedback therapy as it did not result in an improvement in anal manometry pressures or anal incontinence. This may be due to poor compliance, as the women included found it difficult to designate time to use biofeedback [112] (Evidence level 1+).

A parallel two-armed RCT (unblinded) including 109 women with anal incontinence stratified on OASI with primary sphincter repair and hospital affinity found a significant difference in the reduction of St. Mark's scores from baseline to post-intervention in favor of the individualized pelvic floor muscle training group (change in St. Mark's score –2.1 [intervention group] vs −0.8 (control group), *p* = 0.04). The results indicated that individually adapted pelvic floor muscle training reduces postpartum AI symptoms [113] (Evidence level 1−).

A prospective observational study, which was aimed at determining the prevalence of urinary, anorectal, and sexual symptoms arising from OASI within 6 weeks after birth (*n* = 131 women) at their first physiotherapy appointment (immediate postnatal) and 3–6 months (*n* = 96 women) after birth following completion of physiotherapy (intermediate postnatal), showed that physiotherapy did not improve fecal incontinence. They found a significant improvement in pain on defecation (*p* = 0.008), rectal bleeding on wiping (*p* = 0.0233), and women's ability to defer a bowel motion (*p* = 0.0001); however, fecal incontinence did not improve significantly, even with supervised physiotherapy. It is important to note that the prevalence of fecal incontinence at 6 weeks following birth was only 3.2%. The authors concluded that further investigation is required to confirm whether early anal symptomology may be a predictor of long-term adverse outcomes [114] (Evidence level 2−).

A qualitative, inductive descriptive study in 18 women with OASI found that women who suffer from a second-degree birth tear after pregnancy can feel safe when their needs are met. Still, uncertainty is also common when available health care and information are perceived as insufficient. The women also feel uncertainty about what is normal after the tear and how to perform pelvic floor exercises [115] (Evidence level 3).

Access to a physiotherapist is limited in many health care settings, particularly in LMICs. The following can be used as a guide for health care professionals to ensure that women with OASI receive tailored pelvic floor rehabilitation.

Free resources to guide pelvic floor exercises in the postnatal period are available through the IUGA: https://www.yourpelvicfloor.org/pelvic-floor-exercise-videos/.

### Short- and Long-Term Outcome of Primary Repair

#### What Are the Short-Term Outcomes of Primary Repair?

**Clinicians should be aware that women are at risk of wound complications, including infection and dehiscence, following primary repair of OASIs (B).**

Obstetric anal sphincter injuries are susceptible to wound complications as the perineum is an area of high moisture and high surface temperature, with little aeration, which weakens the barrier function of the overlying skin [116]. Also, the risk of wound complications is increased further owing to the proximity to the complex microflora of the surrounding genitourinary and gastrointestinal tract [117].

A systematic review and meta-analysis demonstrated that the incidence of wound infection and wound dehiscence following primary OASI repair is 4.2% (95% CI 1.7–7.5%) and 6.7% (95% CI 3.3–11.0%) respectively [118] (Evidence level 2++).

Obstetric anal sphincter injuries are themselves a risk factor for wound complications following primary repair. One retrospective cohort study of 909 women demonstrated the following independent risk factors for wound complications following primary repair of OASIs.

- OVB (OR 1.76; 95% CI 1.15–2.68) [119].
- Body mass index (BMI; 1-unit increase; OR 1.06; 95% CI 1.01–1.12).
- Smoking (OR 4.04; 95% CI 1.40–12.2).
- Fourth-degree tear (OR 1.89; 95% CI 0.99–3.61).

(Evidence level 2−)

#### What Are the Long-Term Outcomes of Primary Repair?

**Clinicians should be aware that wound complications following primary repair of OASIs may increase the risk of a recurrent OASI in a subsequent birth (C).**

**Women should be advised that up to 24 months following primary OASI repair 82% are asymptomatic (B).**

**Women should be informed that the likelihood of experiencing anal incontinence increases with higher grades of OASI and with time following primary repair (B).**

A cohort study of 411,317 women in Sweden identified that women with an OASI and a wound complication (defined as wound infection, dehiscence, or perineal hematoma within 2 months of childbirth) in the first birth increased the odds of a recurrent obstetric anal sphincter injury (rOASI) by ten-fold (aOR 9.97; 95% CI 6.53–15.24). This remained significant after adjusting for factors associated with OASI in the subsequent birth, including prolonged labor, OVB, fetal malposition, and birthweight ≥4000 g [60] (Evidence level 2+).

A network meta-analysis of studies that used the Sultan classification including 2647 women demonstrated that between 9 weeks and 24 months following primary OASI repair, the overall incidence of anal incontinence was 18.1% and this increased with grade of tear: 3a = 15.6%, 3b = 18.3%, 3c = 20.6%, fourth = 28.4% [120, 121]. The probability of experiencing anal incontinence, fecal incontinence, flatal incontinence, and fecal urgency was highest with fourth-degree tears and lowest with grade 3a tears. St Mark’s Incontinence Score was significantly higher with grade 3b (mean difference [MD] 0.7; 95% CI, 0.3–1.0), grade 3c (MD 1.5; 95% CI 0.9–2.1), and fourth-degree tears (MD 0.9; 95% CI 0.0–1.8) in comparison with grade 3a tears. In addition, grade 3c tears (MD 2.36; 95% CI 1.24–4.49) were significantly higher than those with grade 3b tears. At 3 months, there was no significant difference in the incidence of anal incontinence across the tear groups. However, after 3 months, anal incontinence was significantly higher among those with fourth-degree tears (OR 2.57; 95% CI 1.24–5.29) than among those with grade 3a tears. In addition, anal incontinence was significantly higher among those with fourth-degree tears (OR 2.93; 95% CI 1.37–6.26) than among those with grade 3b tears. Although at 3 months following repair, there was no significant difference in the incidence of anal incontinence across the tear groups, after 3 months, anal incontinence was significantly higher with fourth-degree tears (OR 2.57; 95% CI 1.24–5.29) vs grade 3a and grade 3b tears (OR 2.93; 95% CI 1.37–6.26). (Evidence level 2++).

### Early and Late Complications

#### Wound Dehiscence: Early vs Late Repair

##### How Should Women With Obstetric Anal Sphincter Injury and Wound Dehiscence Be Managed?

**If a woman is experiencing wound dehiscence of the primary OASI repair, referral to a specialist gynecologist or colorectal surgeon should be made for investigations, including EAUS and consideration of conservative or surgical management (GPP).**

**Early repair of a dehisced primary OASI repair can be offered to women (C).**

**In the absence of robust evidence to either support or refute early secondary repair of dehisced OASI wounds, the decision should be tailored to the experience of the clinician and the preferences of the woman (C).**

**Inform women that there is a risk of wound dehiscence, wound infection, and fistula formation following early secondary repair of dehisced OASIs (C).**

**Inform women that the risk of anal incontinence with early secondary repair of dehisced OASIs in comparison with late repair is unknown (C).**

One RCT compared perineal resuturing versus expectant management of dehisced second-degree perineal tears [122]. However, no randomized controlled study exists comparing resuturing versus expectant management in dehisced OASIs (Evidence level 1−).

Traditionally, it is recommended that repeat repair of primary anal sphincter dehiscence should be delayed for 3–6 months to allow neovascularization and resolution of inflammation and infection [123, 124], thereby reducing the risk of wound complications such as infection, abscess, and fistula formation [125]. However, this delayed approach is associated with an increase in maternal morbidity and a reduction in quality of life owing to persistent anal incontinence, pain, and sexual dysfunction whilst awaiting secondary repair [126]. Therefore, the decision to offer surgical management often depends on the clinician’s experience and the woman’s preferences (Evidence level 4).

One review of cohort studies and case series including 96 women identified that most women undergoing early secondary sphincter repair had a satisfactory postoperative recovery with few complications. The most common complication following early secondary sphincter repair included skin dehiscence in 10.3%, wound infection in 5.2%, and fistula formation in 8.2%. Of those that developed a fistula, 50% (*n* = 4) of these required surgical repair, 37.5% (*n* = 3) closed spontaneously, and 12.5% (*n* = 1) were lost to follow-up. The majority of these wounds were fourth-degree tears or rectal buttonhole tears [127]. The risk of these complications, particularly the development of fistulae, must be considered by clinicians when counseling women regarding the management of dehisced OASIs (Evidence level 3).

No RCT exists comparing early repair with later repair of dehisced OASIs. One prospective case–control study compares long-term outcomes (50 months) of 22 women undergoing delayed primary repair, performed more than 72 h after birth or early secondary repair performed within 14 days postpartum, and 19 age-matched and parity-matched women without OASI after vaginal birth. The Wexner score was higher (mean 4.1 vs 1.1; *p* < 0.10) in the study group in comparison with the control group. In addition, symptom bother was significantly higher (mean 1.7 vs 0.3; *p* < 0.01); however, this did not result in a significant change in quality-of-life outcomes [80] (Evidence level 3).

##### Who Should Perform the Early Secondary Repair of Dehisced Obstetric Anal Sphincter Injuries?

**Appropriately trained practitioners should perform early secondary repair of dehisced OASIs, and a colorectal opinion should be considered if the obstetrician is uncertain (GPP).**

**Clinicians should be aware that the optimal timing for early secondary repair is not known (D).**

**Clinicians should avoid undertaking early secondary repair in the presence of obvious active infection (D).**

Early secondary anal sphincter repair is an uncommon surgical procedure and has been reported to be required following 2.6% of primary repairs [119]. Therefore, inexperienced attempts at early secondary anal sphincter repair may further increase the risk of complications such as wound dehiscence, infection, fistula formation, and subsequent anal incontinence. Four observational studies [80, 123, 125, 128] and two case series [127, 129] described senior obstetricians, urogynecologists, and colorectal surgeons performing the procedure. In all cases, a covering stoma was not required. The timing of repair reported in the studies differed and ranged between 2 and 60 days following birth [127] (Evidence level 3).

Wound-healing complications can potentially be minimized with preoperative perineal irrigation and antibiotics. In the four observational studies [80, 123, 125, 128] and two case series [127, 129] describing their experience with early secondary anal sphincter repair, daily preoperative perineal irrigation and antibiotic provision if there was evidence of infection constituted standard practice. However, high-quality evidence is required to inform practice (Evidence level 3).

#### Sexual Dysfunction and Perineal Pain Post-Obstetric Anal Sphincter Injury

##### What Is the Impact of Obstetric Anal Sphincter Injuries on Perineal Pain?

**Women with OASI should be informed that they are likely to experience perineal pain in the first months after birth (B).**

**Women with OASI should be provided with instructions for self-care (GPP).**

A prospective cohort study in 447 parturients assessed the incidence of perineal pain in the first week and 6 weeks postpartum and its association with perineal injury [130]. Although acute perineal pain within 1 week postpartum was associated with OASI (aOR 2.1; 95% CI 1.5 – 2.5), perineal pain was not associated with OASI 6 weeks postpartum [130]. Conversely, a prospective cohort study on 209 women 7 weeks postpartum showed that women with OASI reported significantly more perineal pain than women with second-degree injury or episiotomy [131] (Evidence level 2+).

A prospective cohort study in 3006 women who gave birth for the first time compared perineal pain in women with and in those without OASI [132]. One-month postpartum, 75% of women with OASI reported perineal pain compared with 42% of women without OASI (*p* < 0.001) [131, 132]. At 6 months postpartum, women with OASI were more likely to report perineal pain (12.6% vs 7.3%, *p* = 0.013); however, this difference was not significant after correction in adjusted models [132]. One-year postpartum, perineal pain was more common in women with OASI than in women with no/first-degree injury (OR 3.3; 95% CI 1.1–10) [132]. Also, women with an anal sphincter defect at ultrasound were more likely to experience perineal pain 1 year postpartum (OR 2.3, 95% CI 1.0–5.1) [133]. At an average of 4 years after OASI, 18% of women suffered from chronic pelvic pain (visual analog scale [VAS] score ≥4), and for 8.8% the pain was severe (VAS score ≥7) [134]. Yet, OASI was not associated with pelvic pain 6–11 years after a first birth [135] (Evidence level 2+).

##### What Is the Impact of Obstetric Anal Sphincter Injuries on Sexual Function?

**Clinicians should be aware that anal sphincter injuries negatively affect sexual function in the postpartum period and beyond (B).**

**Clinicians should assess sexual function and address sexual concerns during the postpartum period and at the perineal clinic (GPP).**

##### Resumption of Sexual Intercourse

In a cohort study of 516 postpartum women, OASI was the strongest predictor of postponed resumption of sexual intercourse, defined as beyond 8 weeks postpartum (aOR 5.59; 95% CI 1.59–19.16) [136]. Another prospective cohort study in 199 women with OASI found that 59.8% of women did not resume sexual activity after 12 weeks [137]. Women who were not sexually active 12 weeks after OASI reported more severe symptoms of incontinence (Female Sexual Function Index scores 15.4 ± 12.3 vs 12.0 ± 12.8, *p* = 0.02) and avoidance of sexual activity for fear of incontinence (*p* = 0.003) [137]. After coital resumption, women who resumed intercourse after 12 weeks reported more severe dyspareunia (27.6% vs 17.5%, *p* = 0.29) and more severe symptoms of sexual dysfunction ( Pelvic Organ Prolapse/Urinary Incontinence Sexual Questionnaire 12 scores 38.4 ± 4.1 vs 35.4 ± 5.9) [137]. A prospective cohort study in 377 women giving birth for the first time showed that women with OASI were five times less likely to be sexually active 1 year after childbirth (OR 0.2; 95% CI 0.04–0.93) [138] (Evidence level 2++).

##### Sexual Symptoms 1 Year Postpartum

A longitudinal study in 832 women showed that postpartum symptoms of sexual dysfunction improve between 6 and 12 months postpartum [139]. At 12 months postpartum, nearly half of women with OASI still report dyspareunia compared with 1 in 4 women with no tear or a minor tear (39% vs 6–19%; *p* < 0.05 [136] or 53% vs 25–38%) [140]. A systematic review and meta-analysis from 2021 showed that women with OASI had increased odds for both dyspareunia (OR 1.92; 95% CI 1.47–2.52) and for sexual dysfunction (OR 3.00; 95% CI 1.28–7.03) in the first year after childbirth [141]. Later publications are in line with those findings [142–146]. Additionally, a cohort study on 239 postpartum women showed that women with clinically detected OASIs or undetected anal sphincter defects at ultrasound were more likely to experience dyspareunia 12 months after birth (OR 2.4; 95% CI 1.2–4.9) [133] (Evidence level 2++).

##### Sexual Symptoms in the Mid and Long Term After Obstetric Anal Sphincter Injuries

Three to four years after OASI, almost half of women report dyspareunia, 40% report orgasmic deficiency, and 69% report inadequate lubrication [134]. Four years after childbirth, women with OASI are more likely to experience dyspareunia than women without OASI (OR 2.67; 95% CI 1.44–4.92) [147]. Additionally, some women reported anal leakage during sexual intercourse [148]. Yet, only 1 in 5 women with incontinence sought medical care to address that issue [148]. Four years after OASI, nearly half of women and one-third of their male partners meet criteria for sexual dysfunction [149]. In the couple, both women and men reported infrequency of intercourse to be the principal source of sexual dysfunction [149]. Additionally, women experienced dysfunction related to the domains of desire, arousal, and orgasm, as well as problematic levels of noncommunication and avoidance of sexual activity [149, 150]. In particular, women with grades 3a and 3b reported better sexual function 5 years postpartum than women with grade 3c [151]. Also, fear of incontinence and pain limiting intercourse were reasons for not being sexually active after OASI [134] (Evidence level 2+).

To date, the available literature is not consistent about sexual function decades after OASI. On the one hand, a retrospective case–control study in 109 women found that dyspareunia was associated with OASI 15 years after giving birth (*p* = 0.01) [152]. The authors also found that women with OASI were more likely to report fecal incontinence during intercourse (*p* = 0.005) [152]. However, a retrospective cohort study and two retrospective case–control studies did not find any association between OASI and sexual symptoms several years after OASI [135, 153, 154]. At 25 years, Mous et al. [152] demonstrated that women with OASI were more likely to report dyspareunia in comparison with controls (29% vs 13%, *p* = 0.01) (Evidence level 2+).

**Clinicians should be aware of the negative physical and psycho-social impact of OASI on women’s lives (B).**

**Clinicians should address pelvic floor symptoms and their impact and promptly discuss the available therapeutic options (GPP).**

##### Physical Impact

In a prospective cohort study in 3006 women who gave birth for the first time, those with OASI were more likely to report fatigue (*p* = 0.014) and being unable to do normal activities 1-month postpartum (*p* = 0.015) [132]. Yet, 6 months after childbirth, no difference was found in terms of physical health outcomes [132]. Women with OASI attended the postpartum visit more frequently (86.8% vs 79.9%; *p* = 0.029), but no differences were found in office visits, in emergency department visits, and in procedures or hospitalizations between women with and those without OASI [132] (Evidence level 2++).

##### Body Image

Women with OASI perceived their bodies as “injured,” [155, 156] and reported perceived changes to their genital anatomy and dysfunction [155, 157].

A retrospective study in 422 women after OASI assessed the impact of OASI on body image [157]. Up to 53% of women with OASI reported a change in body image, 33% of women felt less attractive, 19% reported lower self-esteem, and 18% a change in personality owing to the change in body image [157]. Risk factors for reporting change in body image were a perceived anatomical change (aOR 6.11; 95% CI 3.56–10.49), symptoms of anal incontinence (aOR 1.97; 95% CI 1.16–3.36), and forceps extraction (aOR 2.59; 95% CI 1.23–5.43) [157] (Evidence level 2+).

##### Psychological Impact

A systematic review of quantitative and qualitative studies examined women’s experience after OASI, including psychological sequelae and implications for future pregnancies [158]. The first 2 months after OASI were a “worse nightmare than expected” for women with persistent problems of severe pain and incontinence [159]. Next to the persistent pain and the physical and psychological limitations, women’s expectations of early motherhood were “crushed” [155, 159, 160], with a negative impact on their commitment to parenting tasks [161] (Evidence level 1+).

Women with OASI reported significantly more symptoms of post-traumatic stress (aOR 2.73; 95% CI 1.27–5.86) [161, 162] (Evidence level 3).

Yet, there is no consensus on the association between OASI and postpartum depression. Most studies found that women with OASI reported symptoms of postpartum depression more often (OR 4.67; 95% CI 1.37–15.92) [134, 146, 163]. Other authors did not find significant differences [132, 164]. When a subgroup analysis was performed in women with low resilience (measured using the Sense of Coherence 29 scale), those with OASI reported higher odds for postpartum depression (aOR 5.5; 95% CI 1.2–26) [164] (Evidence level 2++).

Also, women with OASI reported reduced interest in intimacy and grief over the loss of functional sexuality with their partner [165], as well as anxiety [134, 166] and fear about future pregnancies and childbirth [156, 157, 165, 166] (Evidence level 2+).

##### Quality of Life

Obstetric anal sphincter injury seems to have a negative effect on the quality of life of women after childbirth (36-Item Short Form Survey adjusted MD −6.87; 95% CI −9.93 to −3.83) [167]. Yet, the current literature is not always consistent; studies are heterogeneous in terms of the tools for quality-of-life assessment and timing after childbirth (Evidence level 3).

A longitudinal study in 35 women with OASI found that quality of life significantly improved over the first 6 months postpartum, in particular for the domains of physical functioning, vitality, and social functioning [145]. Nine weeks after primary OASI repair, women with OASI grades 3c and 4 had lower quality-of-life scores when compared with women with OASI grades 3a and 3b. Differences were found in the “mean role” (*p* = 0.01), “physical function” (*p* = 0.02), and “severity measures” (*p* = 0.01) domains of the Manchester Health Questionnaire [168]. Conversely, no significant difference was found between women with OASI grade 3c and women with OASI grade 4, as well as between women with OASI grades 3a and 3b [168]. At 6 months, no difference was found between women with and those without OASI in terms of quality of life [145] (Evidence level 2).

One year postpartum, women with OASI were reporting more often that the injury was affecting their daily life (OR 18; 95% CI 5.1–59) and that they were more likely to experience severe perineal pain (OR 3.3; 95% CI 1.1–10) and urine and anal incontinence affecting lifestyle (OR 4.6; 95% CI 1.5–14/OR 23; 95% CI 2.5–21.3) [142] (Evidence level 2+).

Within 2 years after OASI, no correlation was found between OASI grade and quality of life [169]. Four years postpartum, 30% of women with OASI reported that their anal incontinence affected their daily life compared with 10% in women without OASI. Also, women with OASI grade 3c and grade 4 reported a worse quality of life than women with OASI grades 3a and 3b [151]. Additionally, negative impacts on sexual functioning decreased quality of life slightly in 22.5% of women with OASI, whereas 8.5% women with OASI stated a moderate or strong negative impact on quality of life [170] (Evidence level 2−).

A decade after childbirth, 1 in 3 women with OASI reported that their quality of life was affected, which was associated with a Wexner score ≥2 (sensitivity 0.94; 95% CI 0.92–0.96/specificity 0.85; 95% CI 0.84–0.87) [170]. Nearly 1 in 2 women with incontinence after OASI reported a change in quality of life in all fields of the Fecal Incontinence Quality of Life questionnaire [171] (Evidence level 2−).

### Assessment and Investigation Tools After Obstetric Anal Sphincter Injuries

**Women with a clinical diagnosis of OASI require an assessment by an experienced provider consisting of history, physical examination, and investigations (D).**

Women with a clinical diagnosis of OASI should undergo postpartum assessment by an experienced health care provider, including a comprehensive history, validated questionnaires, physical examination, and appropriate investigations. Key components of history and physical examination are outlined in the Canadian 2025 guideline and presented in Appendix 2. Ideally, this evaluation occurs in a dedicated clinic to monitor postpartum recovery and detect any sequelae associated with OASI. Counseling addresses pelvic floor rehabilitation, options for the mode of subsequent birth, and strategies to prevent pelvic floor disorders in the future. Although the timing of follow-ups varies among dedicated clinics, it is reasonable to initiate assessment within 1–2 weeks postpartum to screen for early wound complications [172]. Subsequent follow-up visits may be scheduled at specific time intervals (e.g., 6 weeks, 3 months) or tailored according to the evolution of symptoms [173] (Evidence level 4).

For pregnant women with a history of OASI, evidence regarding the optimal timing of assessment is limited. However, this timing should be individualized, considering each woman’s unique clinical and social circumstances and local protocols. Expert opinion suggests that assessment in the third trimester (after 28 weeks’ gestation) is appropriate, taking into account several factors: the risk of spontaneous abortion and potential termination of pregnancy; the time required to arrange a specialist assessment and complete necessary investigations; the importance of allowing adequate time for a woman to consider her options and make an informed decision regarding mode of subsequent birth; and the logistical planning needed if an elective CS is chosen.

**Which investigations can be used to assess the anal sphincter complex in women with OASI?**

**Advanced: EAUS is considered the gold standard for identifying structural defects, whereas anorectal manometry (ARM) is the preferred modality for objectively evaluating the function of anal sphincter complex (B).**

**Essential: If EAUS is not available, 2D-transperineal ultrasound (TPUS) or 3D-TPUS may be used as a screening tool. Referral for EAUS is recommended when a residual anal sphincter defect is identified for confirmation of diagnosis (B).**

**Desirable: If ARM is not available for functional assessment, digital rectal examination may be used together with EAUS, 2D-TPUS, or 3D-TPUS (B).**

Anorectal investigations in women with OASI should ideally include EAUS and ARM, as these are the most accurate tools for assessing anal sphincter structure and function [174]. In centers where EAUS and ARM are not available, alternative diagnostic modalities should be considered based on locally available resources. The objectives of anorectal investigations are as follows:

1. To verify the clinical diagnosis of OASI.
2. To evaluate both the structural integrity and functional capacity of the anal sphincter complex.
3. To develop individualized recommendations regarding the mode of subsequent birth.
4. To guide management of anorectal symptoms, including planning for secondary anal sphincter repair or sacral neuromodulation (SNM).
5. To counsel patients regarding their risk of developing anorectal symptoms in the future.

The following table shows the investigative tools for women with prior OASI:

**Table:** 

Structural assessment of the anal sphincter complex	Functional assessment of the anal sphincter complex
• EAUS^a^	• ARM^d^
• 2D-TPUS (introital ultrasound)^b^	• DRE (may utilize DRESS [175]^e^
• 3D-TPUS^b^	
• MRI	
Avoid the use of DRE alone for structural assessment^c^	

*ARM* anorectal manometry, *DRE* digital rectal examination, *DRESS* Digital Rectal Examination Scoring System, *EAS* external anal sphincter, *EAUS* endoanal ultrasound, *IAS* internal anal sphincter, *MRI* magnetic resonance imaging, *2D-TPUS* two-dimensional transperineal ultrasound, *3D-TPUS* three-dimensional transperineal ultrasound

^a^Gold standard for structural assessment of the anal sphincter complex with sensitivity and specificity of 100% for identifying an EAS defect and sensitivity of 100% and specificity of 95.5% for identifying an IAS defect [176]

^b^May be used as a screening tool for the structural assessment of the anal sphincter complex with referral to EAUS if the anal sphincter defect is identified for confirmation of diagnosis. 2D-TPUS and 3D-TPUS have a high negative predictive value (2D-TPUS: 86% for EAUS, 93% for IAS; 3D-TPUS: 85% for EAS, 93% for IAS) and a low positive predictive value (2D-TPUS: 50% for EAS, 63% for IAS; 3D-TPUS: 51% for EAUS and 37% for IAS) for the detection of a residual anal sphincter defect [177]

^c^The use of DRE alone for the assessment of the structure of the anal sphincter complex and the diagnosis of residual EAS defects should be avoided owing to sensitivity of 12–82% and specificity of 32–55% for the diagnosis of EAS defects observed on EAUS [178–180]

^d^If ARM is not immediately available, it may be performed during a subsequent pregnancy or prior to the surgical management of anorectal symptoms

^e^DRE may be used together with EAUS, 2D-TPUS, or 3D-TPUS; however, it should be noted that no validation studies have yet been conducted [179, 181]

For LMICs, where access to EAUS and AM may not be available, there will be difficulty in assessing anal sphincter morphology and function adequately. Symptom assessment alone is flawed as symptom prevalence increases with time. Mous et al. [152] demonstrated in their 25-year longitudinal study of 171 women with OASI that symptom prevalence increased from 38% to 61%. Moreover, in women with no symptoms, by the 15- and 25-year follow-ups, 43% and 11% respectively had developed symptoms. Okeahialam et al. [174] demonstrated in their 20-year retrospective analysis, including 607 women, that 91% of asymptomatic women with a grade 3a tear (*n* = 228 women) would have normal EAUS and AM, meaning that a mode of birth recommendation supporting vaginal birth could be made.

## Section 4: Long-Term Management of Patients with Obstetric Anal Sphincter Injuries

**Subspecialty clinics dedicated to the care of patients with OASI provide an opportunity for streamlined early, comprehensive care in this high-risk population (C).**

Dedicated subspecialty clinics were first reported over 20 years ago in the UK [182]. Since that time, these dedicated clinics have grown in prevalence. Ten subspecialty clinics internationally were identified in a 2019 literature review, but this is likely an underestimation given the vast number of established clinics in the USA and abroad. [183, 184].

Given that OASI is associated with direct pelvic floor injury and an increased risk of short- and long-term pelvic floor sequelae, complications such as wound infection and dehiscence, and a significant negative impact on quality of life and mental health, offering patients a consultation with a pelvic floor specialist early in the postpartum period is ideal. This visit is an opportunity to evaluate healing, identify symptoms, educate patients, and provide anticipatory guidance on recovery and future births. A variety of care bundles have been described in the literature, which highlight the experience of care during and after an OASI [184, 185].

Guidelines on post-OASI management from professional organizations internationally are heterogeneous and sparse [186]. Regarding the timeline for follow-up, the American College of Obstetricians and Gynecologists (ACOG) and Australian guidelines recommend evaluation between 1 and 2 weeks after birth. In contrast, other organizations, such as the RCOG and German guidelines, recommend follow-up at 6–12 weeks and 12 weeks respectively [186, 187]. Although some organizations do not make specific recommendations regarding the type of provider the patient should follow up with, the RCOG supports patients seeing a clinician with a “special interest in OASI,” Australian guidelines support “health care professional with experience in pelvic floor health,” and German guidelines suggest follow-up with a gynecologist or coloproctologist [186, 187].

Previous publications have highlighted how to establish a subspecialty clinic [188–190]. A variety of models have been described, including differing provider specialties, consolidated versus staged assessments, telehealth versus in-person evaluations, specific versus expanded patient populations, and varying management protocols [189]. Care provided by these clinics has been described in the literature, the longest of which highlights 11 years of outcomes [189].

Importantly, these clinics provide a variety of benefits to patients and providers alike. First, patients have an opportunity to have an evaluation for symptoms such as bowel control issues, bladder control issues, pain, and dyspareunia. Previous publications have demonstrated poor provider comfort with OASI care, as well as a lack of patient willingness to volunteer symptoms such as bowel or bladder incontinence [191, 192]. This results in patients “suffering in silence” [192]. Some clinics also evaluate for postpartum depression and issues with lactation, as this may be the first interaction that a patient has with a provider post-birth. Further, patients can have their laceration evaluated. Previous literature has reported up to a 25% wound breakdown rate after OASI, and this is often within 2 weeks of birth [12]. Fistulae and anal sphincter separation, although not common, are significant sequelae that can be identified on examination. Ancillary testing, such as EAUS and ARM, can be utilized if available in the clinic or coordinated with other health care professionals. Education on the tear incurred, including risk factors (both modifiable and nonmodifiable), can be provided, and implications for future births can be discussed. Referral to pelvic floor physical therapy for rehabilitation can also be facilitated. Surgical intervention after OASI is necessary in 9% of patients. With dedicated follow-up, these candidates can be identified appropriately, and surgery can be performed in a timely manner, within 2 to 3 weeks of birth [80, 128, 175]. Referring obstetric providers have also reported great satisfaction with the presence of a dedicated OASI clinic and a positive impact on the care that these clinics provide to patients [193]. Last, these clinics provide a vital opportunity to advance the provision of care to this vulnerable population through high-quality education, counseling, and interventions aimed at minimizing the risk of long-term sequelae from OASI and fueling novel research to inform the care for patients with OASI (Evidence level 2+).

**Both EAUS and ARM can be used to help to differentiate laceration sequelae and inform discussion on future births (C).**

The incidence of bowel control issues in the short and long term after OASI is significantly higher than for those with no or lesser degree lacerations [194]. It is crucial that the degree of laceration is accurately identified so that an appropriate repair can be performed during the initial procedure. Under-classification, which has been shown to occur in over 10%, results in poorer bowel control [195]. Studies have demonstrated the utility of ultrasound to supplement vaginal and rectal examinations in the birth suite [60]; however, this practice has not been widely adopted, likely because of its expense, required expertise, and limited availability.

During a postpartum evaluation, although a detailed examination, including inspection of the perineal and anal complex, as well as a digital rectal examination (DRE), can be informative, these also have limitations, with reduced sensitivity compared with diagnostic modalities [179]. Ancillary testing with EAUS and ARM can be helpful when the examination findings are ambiguous, the patient’s symptoms are persistent, or to guide further treatment planning, especially if surgery is being considered. EAUS provides complementary information on the anatomy and integrity of the sphincter complex, whereas ARM provides information on its function. Although some controversy exists in the literature, endosonographic anal sphincter disruption, anal incontinence symptoms, and abnormalities on ARM postpartum after OASI have been associated with the subsequent development of fecal incontinence [196, 197].

Similar to guidelines on post-OASI management, there is a paucity of guidance on imaging for this patient population [186]. RCOG guidelines recommend both EAUS and ARM to help to differentiate laceration sequelae and inform discussion on future births, including that if the patient has bowel control issues, or has abnormalities on EAUS and ARM, a CS should be considered [187]. German and Queensland guidelines support EAUS being utilized in symptomatic patients only [186]. Other international organizations make no recommendation regarding sphincter complex imaging [186].

Three-dimensional EAUS is recognized as the gold standard diagnostic modality for evaluation of the sphincter complex. Studies of sphincter complex integrity after primary obstetric repair after OASI reveal persistent defects in over 90% of patients [3]. A recent meta-analysis found this to be lower, at 55% [82]. As previously mentioned, these persistent defects increase the risk of long-term bowel control issues, which is why experts support the use of routine EAUS in high-risk groups within 6–12 weeks postpartum [198]. The diagnosis of a residual defect has been defined as discontinuity of the EAS ≥30° (>1 h) in at least two-thirds of the length of the anal sphincter [179]. Although EAUS has good intra-observer reproducibility, it has not been widely adopted owing to the expense of the equipment, invasiveness, and the technical training required. TPUS has also increased in popularity, likely because of less discomfort for patients and its ability to provide similarly informative images of the sphincter complex without distorting the anal canal [198]. Because the probe is the same as that used with routine obstetric imaging, the equipment is more accessible and may be associated with less patient discomfort than EAUS. An EAS defect of ≥30° (>1 h) on ≥4 of 6 slices has been defined as the criterion for persistent injury using four-dimensional transperineal ultrasound [198].

Anorectal manometry is another tool available to help to evaluate patients after OASI. This involves utilizing a probe to evaluate maximum resting and maximum squeeze pressures. The difference between these two measurements is the incremental maximum squeeze pressure, which has been shown to correlate with EAS function [197]. A more recent study by Trieu et al. identified a cut-off of 59 mmHg for baseline resting pressure as a predictor of long-term fecal incontinence after OASI [196]. Although ARM is used more broadly in the fields of gastroenterology and colorectal surgery, owing to the increasing support for its role in evaluating sphincter complex function, partnering with these specialties may be important to help to direct patient care and inform counseling (Evidence level 2+).

## Section 5: Risk Factors, Prediction, and Prevention

### Can Obstetric Anal Sphincter Injuries Be Prevented?

**Antenatal perineal massage in the third-trimester reduces the risk of OASI (A).**

**Perineal massage during the second stage of labor can reduce the risk of OASI (A).**

**Warm compression during the second stage of labor reduces the risk of OASI (A).**

**Perineal protection at crowning can be preventive (C).**

**Clinicians should consider seeking support from a second clinician to assist with perineal prevention techniques (B).**

**Clinicians should be aware that care-bundle initiatives can be implemented to reduce the incidence of OASI (B).**

#### Antenatal perineal massage:

Third-trimester antenatal perineal massage before vaginal birth has been suggested as a way of preventing significant perineal tears. The Cochrane review [199] of four studies (2947 women) analyzed nulliparous and parous women separately and showed no significant difference with regard to OASI rate. However, the more recent meta-analysis of seven studies (3069 women) demonstrated that perineal massage performed by pregnant women or their partners within 4–6 weeks before birth was associated with a 64% reduction in the incidence of OASI (RR 0.36; 95% CI 0.14–0.89) [200] (Evidence level 1−).

#### Perineal massage in the second stage of labor: 

Perineal massage in the second stage of labor can be performed by clinicians using a water-based lubricant, between and during pushing and for between 5 and 15 min. One meta-analysis of five studies (2487 women) found that the use of perineal massage reduced the rate of OASI by 51% (RR 0.49; 95% CI 0.25–0.94 [201] (Evidence level 1−).

#### Warm compress in the second stage of labor: 

Using a warm compress on the perineum during the second stage of labor has been suggested to improve the stretch and elasticity of the perineum. The warm compress can be held on the perineum continuously during and between contractions. Careful attention should be paid to the temperature of the compress to prevent inadvertent injury to the woman. A Cochrane review [202] of four studies (1799 women) found that warm compresses significantly reduced the risk of OASIs by 54% (RR 0.46; 95% CI 0.27–0.79). In addition, the more recent meta-analysis by Sun et al. [203], evaluating the effect of warm compress in primiparous women alone, including four studies (526 women), demonstrated that this intervention reduced the risk of OASIs by 66% (RR 0.34; 95% CI 0.20–0.57) (Evidence level 1++).

#### Manual perineal protection (MPP): 

Manual perineal protection (MPP): During crowning, the perineal body undergoes a significant increase in tension. It has been shown that the posterior fourchette is mostly affected, particularly anteroposteriorly in the midline [204, 205]. MPP is aimed at reducing tension on the perineal body by dispersing tension over a wider surface area [204, 205]. This can be reduced by 40% when using the dominant hand to apply pressure to the posterior fourchette with the thumb and index finger [205] (Evidence level 2+).

Two manual perineal techniques are described in the literature that utilize this technique, including the Finnish and Viennese techniques [206]. In both techniques, the nondominant hand is placed on the fetal occiput to control the speed of birth, whereas the dominant hand applies pressure to the perineum, either with the thumb and the index finger [207] or the palm [208] whilst approximating them to reduce perineal stretch [206]. To our knowledge, there are no RCTs comparing the two techniques.

The systematic review by Bulchandani et al. [209] of three RCTs (6647 women) and three non-RCTs (74,744 women) demonstrated that within the RCTs, MPP was not associated with a reduction in OASI. However, within the observational studies, MPP was associated with a 55% reduction (RR 0.45; 95% CI 0.40–0.50). In addition, in all the RCTs included, the impact of MPP on OASI rates was not a primary outcome, and there was a lack of technique standardization [209] (Evidence level 1−).

The effect of MPP, particularly within the context of a care bundle, has been shown in interventional studies to have positive effects and should be promoted [11, 16, 210] (Evidence level 2++).

In LMICs the preventative techniques described above can also be employed to reduce the risk of OASI. A prospective study of 240 women in Uganda found that using perineal support at the time of birth reduced the odds of OASI by 97% (aOR 0.03; 95% CI 0.01–0.12) [211] (Evidence level 2+).

The presence of two clinicians during the active second stage to aid interventions to prevent perineal trauma, including warm compress and MPP, has been shown to reduce the risk of OASI in primiparous women. The multicenter RCT by Edqvist et al. [212] found that the odds of OASI reduced by 31% (aOR 0.69; 95% CI 0.49–0.97). However, it is important to note that women and clinicians were not blinded to group allocation and the study was underpowered (Evidence level 2+).

#### Care bundle initiatives: 

Care bundle initiatives: Countries including the UK, Norway, and Denmark have found from large multicenter prospective cohort studies that the implementation of perineal protection initiatives and care bundles can reduce the incidence of OASIs [11, 16, 210].

The RCOG-RCM OASI Care Bundle was implemented in 16 maternity units in the UK and included antenatal education, MPP, use of an MLE if clinically indicated, and systemic vaginal and rectal examination to assess for anal sphincter injury after birth. Implementation of this care bundle for 55,060 vaginal births (79% spontaneous and 21% operative) was associated with a 20% reduction in the risk of OASIs (aOR 0.80; 95% CI 0.65–0.98), with no effect on CS or episiotomy rates [11] (Evidence level 2++).

In Norway, their care bundle was implemented in four maternity units and included recommending vacuum-assisted birth instead of forceps-assisted birth, MLE instead of median episiotomy if clinically indicated, controlled birth of the fetal head, and MPP. Implementation of this care bundle for 40,152 vaginal births reduced the rate of OASI from 4–5% to 1–2% (*p* < 0.001) over 3 years [16] (Evidence level 2++).

In Denmark, a care bundle was implemented in one maternity unit and included good communication with the woman, visibility of the perineum at crowning, controlled birth of the fetal head, and MPP. Implementation of this care bundle for 1622 vaginal births reduced the risk of OASI by 52% (RR 0.48; 95% CI 0.3–0.76) [210] (Evidence level 3).

Care-bundle initiatives like the OASI Care Bundle are well accepted by women. A survey of 1208 women found that the Care Bundle did not have any negative effects on their birth experience. For example, of the 377 women who recalled receiving MPP (31.2%), 203 (53.9%) felt that it was supportive, 97 (25.7%) did not mind the sensation, and 51 (13.5%) felt that it was uncomfortable but understood its importance. In addition, of the 664 women who recalled having a rectal examination (55.0%), 281 (42.3%) were happy to be examined, 335 (50.5%) found it uncomfortable but understood its importance, and 20 (3.0%) did not understand its purpose [213] (Evidence level 3).

### What Risk Factors Are Associated with Obstetric Anal Sphincter Injuries?

**Maternal birth position is not clearly associated with the risk of OASIs (B).**

**Although maternal birth position is not associated with the risk of OASIs, birthing positions that allow additional interventions to prevent OASIs (e.g., MPP or warm compresses) are warranted (D).**

**Fetal malposition, occipito-posterior in particular, is associated with an increased risk of OASIs, both directly and through an increased risk of the need for an OVB (C).**

**Correction of fetal malposition, in both spontaneous and OVB, decreases the risk of OASIs and should be pursued during the second stage of birth after recognition (C).**

**Both forceps- and vacuum-assisted births increase the risk of OASIs. However, this risk is significantly higher with forceps (C).**

**If the obstetric situation permits use of either instrument, the vacuum extractor should be the instrument of choice with respect to the prevention of OASIs (C).**

**Water birth is not associated with an altered risk of OASIs (C).**

**Mediolateral/Lateral episiotomy (MLE/LE) should be used selectively in women with spontaneous vaginal delivery (SVD). There is no place for its routine use in SVDs (A).**

**Nulliparous women may benefit from the use of MLE with regard to the occurrence of OASI, especially in the case of accumulation of risk factors, e.g., high fetal birthweight and prolonged second stage of labor. This benefit should be balanced against possible drawbacks of the use of MLE/LE (B).**

**Use of MLE/LE in multiparous women in SVDs is only warranted in special cases and should be discouraged in general (A).**

**Mediolateral/LE/LEs are associated with a lower risk of OASIs in OVB in nulliparous women, in which the association in forceps-assisted births is stronger than in vacuum-assisted births. Liberal use of MLE/LE in these women is advocated (A).**

**Mediolateral/LEs are associated with a lower risk of OASIs in OVB in multiparous women, but this association is less pronounced in vacuum-assisted births than in forceps-assisted births. This benefit should therefore be balanced against possible drawbacks of the use of MLE/LE when this is considered (B).**

**The angle of the MLE is associated with a risk of OASI. Clinicians should ensure that the angle is 60° from the midline at crowning (C).**

**Use of specially designed devices to achieve adequate angulation of MLEs is recommended as its use results in a better-quality episiotomy and in a reduced risk of OASI (C).**

**The risk of OASIs for women with VBAC is significantly higher than that for nulliparous women (possibly dependent on the indication for the prior emergency CS) (C).**

**Mediolateral episiotomy is associated with a significantly lower OASI risk during VBAC and may be considered in these women as a preventive measure (C).**

**Obstetric anal sphincter injuries are associated with a shorter perineal length, but clear preventive measures have not yet been established (C).**

**Female genital mutilation/cutting is associated with an increased risk of OASIs and of perineal lacerations in general. This association exists both for women delivering in Africa and for women delivering after migration to high-income countries (C).**

**Mediolateral episiotomy in addition to intrapartum deinfibulation, where indicated, should be considered as a protective measure for women with female genital mutilation/cutting (FGM-C) if the obstetrical circumstances allow proper repair and healing (C).**

#### Maternal Birth Position

There is currently no definitive evidence that maternal birth position directly influences severe perineal lacerations during childbirth [214–217]. In a systematic review and meta-analysis of 50 studies, 13 studies of a total of 3040 women were included on maternal upright positions compared with supine and there was no significant difference in the occurrence of perineal lacerations [216] (Evidence level 1−).

This finding is consistent with a Cochrane review that showed no clear differences in OASIs between upright and supine maternal birth positions [215] (Evidence level 1+).

Studies on maternal birth position are heterogeneous and have not fully explored the effects of all birthing positions on OASIs or risk factors for OASIs. In a Cochrane review, upright (compared with supine) birthing positions were associated with a reduced duration of the second stage of labor compared with supine position by only 6 min, which is unlikely to be clinically relevant. However, there was also low-quality evidence to suggest a reduction in OVBs and episiotomies, which are also associated with OASIs [215]. Another comprehensive meta-analysis of multiple birthing positions including 1985 laboring women concluded that flexible sacrum positions were associated with a reduced duration of the second stage of labor by 21.12 min [218]. Additionally, a Cochrane review of maternal birth position during labor to correct fetal malposition concluded that all-fours and lateral positions showed no evidence of impact on maternal or fetal outcomes, but that more research is needed regarding other postures [219] (Evidence level 1−).

Although maternal birth positions may not effectively prevent OASIs, they do not show evidence of harm either. Additional research is needed regarding outcomes of different maternal birth positions, as well as to determine if there is an impact on risk factors for OASIs, such as duration of the second stage of labor, operative birth and episiotomy rates, controlled birth at the perineum, and fetal malposition.

#### Fetal Position

Fetal malposition is an independent risk factor for OASIs [214, 220–224]. In a meta-analysis including 12 studies and 369,427 patients, occiput posterior position carried an RR of 2.73 for severe perineal trauma (95% CI 2.09–3.58) [220]. Occiput posterior position is also associated with a prolonged second stage of labor and OVB, which may compound the risk of OASI [225–227] (Evidence level 2++).

In an RCT of manual rotation from occiput posterior to anterior positions, the second stage of labor was shorter, but OVB, OASIs, and episiotomy were not significantly different between groups [228] (Evidence level 1+). However, there is evidence that manual rotation from occiput posterior to anterior in the setting of OVB can reduce the risk of OASIs within this high-risk group. In a case–control study of 249 women undergoing OVB, successful manual rotation from occiput posterior to anterior position was associated with a significantly lower proportion of OASIs (22% vs 75%, *p* = 0.01) [229]. In a retrospective cohort study of manual rotation from occiput posterior to anterior in the setting of forceps-assisted birth, the risk of severe perineal lacerations decreased from 43.4% to 24.3% (*p* = 0.02) [224] (Evidence level 2+).

Furthermore, in a prospective cohort of 111 women delivered by OVB after failed manual rotation followed by an attempt at instrumental rotation, persistent occiput posterior position was associated with a higher proportion of OASIs (24.5% vs 1.7%; *p* < 0.001) and a higher likelihood of anal incontinence at 6 months (30% vs 5.5%; *p* < 0.001) compared with those successfully turned to the occiput anterior position by instrumental rotation [230] (Evidence level 2−).

The results of these studies suggest that fetal malposition, although also associated with prolonged labor and OVB, is an independent and modifiable risk factor for severe perineal lacerations and OASIs. Manual rotation and instrumental rotation may be effective at modifying these risks, whereas maternal positioning in pregnancy and during labor does not have an apparent benefit [219, 230]. Further investigation on the effectiveness of less invasive interventions that correct fetal malposition are needed.

#### Operative Vaginal Birth

In general, two types of OVB are in use globally: vacuum- and forceps-assisted birth. There is a distinct variation between countries regarding the preferred instrument when an OVB is indicated. In many countries with an Anglo-Saxon obstetrical tradition, the forceps is commonly used. In most countries on the European continent the vacuum extractor is predominantly used in the case of OVB.

It is accepted that both types of OVB are associated with an increased risk of OASIs compared with spontaneous vaginal birth (SVB). This is demonstrated by the results of large observational studies based on large national obstetrical databases [1, 231] (Evidence level 2++).

The most recent Cochrane review Instruments for Assisted Vaginal Birth of 2021, containing nine RCTs with almost 2500 women reported an almost doubled risk of OASIs with the use of forceps compared with vacuum-assisted birth (aOR 1.83; 95% CI 1.32–2.55) [232]. It should be noted that 8 out of 9 RCTs in this review were published before 2010 (Evidence level 1++).

However, the conclusion of this review is in line with more recent observational studies from varying countries. A recent observational study from Norway describing the OASI risk during instrumental vaginal birth over a period of 18 years confirmed this almost doubled risk with the use of forceps compared with vacuum-assisted extraction (aOR 1.92; 95% CI 1.79–2.05) [233]. In a large observational study from the UK containing 1,035,253 singleton, term, cephalic, first births, rates of OASIs were much higher in forceps-assisted births, with or without MLE (6.1% and 22.7% respectively) compared with vacuum-assisted births with or without MLE (2.3% and 6.4% respectively) [1]. This risk difference between the two types of instruments was confirmed in a large Dutch observational study containing data on almost 171,000 OVBs [23] (Evidence level 2++).

In the latest RCOG Green-Top Guideline (GTG) no. 26 on Assisted Vaginal Birth, a guideline with great international impact, concerns are raised with regard to the achievement of vaginal birth with vacuum assistance [234]. In the trials discussed in this guideline, failure rates of 17% to 36% have been reported with the use of different vacuum devices. Critical appraisal of these trials, however, shows that this failure rate is, amongst other possible factors, highly dependent on the type of vacuum cup used. Two UK-based trials compared Kiwi cups with “conventional” cups and in these trials 50% of women’s attempts to deliver were performed with a Kiwi Omnicup, 33% with a Silastic cup, and 17% with metal cups [235, 236]. The failure rates with disposable cups ranged from 26% to 34%, compared with 17% to 21% for conventional cups. In a third trial from France comparing use of a disposable cup with a metal cup, failure rates of 35% and 7.1% respectively were reported [237]. The results of these trials were in contrast to a fourth trial from Papua New Guinea that reported low rates of failure of 2% for the Kiwi Omnicup and 6% for the Bird metal cup [238] (Evidence level 1+).

Authors of an opinion paper published in 2021 state that the failure rate of vacuum extraction appears to be much lower in countries with possibly a stronger tradition of the use of vacuum extraction, and in this possibly more experience, as in these countries the use of a second instrument or CS after a failed OVB attempt is only rarely registered [239]. Observational studies addressing the risk of failure of vacuum extraction from various countries confirm this statement, reporting failure rates of 4–8%. These rates suggest that it might be possible to achieve an acceptable rate of vaginal birth with vacuum extraction without an unwanted rising rate of CS in the second stage of birth (Evidence level 2+).

Possibly, international cooperation and supervised training of vacuum extraction may be the key to overcoming the concern for failure of vacuum extraction by exchanging knowledge and skills and in this way inducing a change of obstetrical tradition and culture.

#### Waterbirth

A systematic review and meta-analysis of six RCTs including 706 patients showed no significant association between episiotomy or perineal lacerations and water birth [240]. Similarly, an updated meta-analysis that also included non-RCTs confirmed an overall low proportion of patients with OASIs and no significant difference between childbirth in water (1.2%; 669 out of 54,384) versus on land (1.72%; 4017 out of 233,416) [241] (Evidence level 1++).

In a recent large observational study describing a cohort of women from the UK with an obstetrical low risk, no difference was again found between water birth and birth out of water [242]. However, the rates of OASIs in this cohort of women, especially in nulliparous women (both in and out of water) were remarkably high (4.8% and 5.3%) (Evidence level 2+).

#### Episiotomy

Episiotomy is one of the most frequently performed interventions in obstetrics but also one of the most debated. In the past, episiotomy was performed quite easily without proper evidence of its efficacy but in the last decades its use has declined considerably.

One of the issues with addressing the evidence for episiotomy as a possible protective intervention for the occurrence of OASIs is the type of evidence used and accepted. Most national guidelines are solely based on the results of randomized trials, as is the most widely used source of evidence, the Cochrane Database of Systematic Reviews.

However, this may cause a skewed view of this subject. An RCT may be the best way of comparing two treatments for a certain condition, but episiotomy is not a treatment for impending OASIs. In fact, it should be seen as a risk-modifying factor for the occurrence of OASIs and in this, evidence from large observational studies, preferably based on well-established multicenter or national databases, should also be taken into account.

Furthermore, published RCTs have shown that only different approaches toward the use of episiotomy can be addressed in this type of study, rather than the absolute role of (mediolateral or lateral) episiotomy itself. In RCTs the policies of routine or liberal use of episiotomy are usually compared with the policy of restricted use or “no episiotomy.”

A second issue to address is the type of episiotomy used to prevent OASIs. Kalis et al. have described the different types of episiotomies mentioned in the medical literature and have shown that, quite commonly, the type of episiotomy used in the described studies is only poorly, if at all, described [243]. The most commonly described types were median/midline and mediolateral episiotomies. In the past decade, it has become clear that midline episiotomies are associated with a significantly higher risk of OASIs in both spontaneous and OVB, and therefore their use is commonly discouraged.

For the role of episiotomy as a possible protective intervention for OASIs we have separated this section into two: use of episiotomy in SVBs and use of episiotomy in OVBs.

#### Episiotomy in Spontaneous Births

The first trial conducted, and to date one of the best, addressing the effect of episiotomy on the occurrence of OASIs was the study of the Argentine Episiotomy Trial Collaborative Group in *The Lancet* in 1999 [244]. In this trial the routine use of MLE was compared with its selective use in women delivering for the first or second time with severe perineal trauma as the primary outcome. More than 2600 women were enrolled in this trial and routine use (82.6%) showed a slightly higher risk of severe perineal trauma (1.5% vs 1.2%) compared with selective use (30.1%). The final conclusion of this trial was that a policy of routine episiotomy should be abandoned and rates above 30% cannot be justified.

The most recent Cochrane review on this subject dates from 2017 and describes 12 studies [245]. One of these 12 studies described the results of a trial in women with OVB only, whereas 7 of the remaining trials also included OVBs without a clear separate analysis of women with spontaneous births only. All trials follow the design as described above; selective versus routine episiotomy or the clinicians common practice for the use of episiotomy. The prevalence of episiotomy use in the selective groups varied between 8% and 59%, whereas these rates in the routine groups varied between 51% and 100%. Furthermore, two studies were included with the use of midline episiotomies, which may give a skewed result from the final result, and most studies included both nulliparous and multiparous women. The outcome for severe perineal trauma was that episiotomy was associated with a relatively small protective effect, with a risk-ratio of 0.70 (95% CI 0.52–0.94). When subdivided according to the difference in episiotomy rate of more or less than 30% in the described trials, it was clear that this effect was only significant in trials with an episiotomy rate difference of more than 30% (RR 0.55; 95% CI 0.38–0.81). Separate analysis according to parity showed that this protective effect was only present in nulliparous women (RR 0.68, 95% CI 0.50–0.93). Analysis according to the type of episiotomy used showed that MLE was no longer associated with a protective effect for OASIs (RR 0.62; 95% CI 0.37–1.04), although no separate analysis according to parity was described for women delivered using MLE. This Cochrane review reflects the difficulty of addressing the effect of episiotomy with regard to the occurrence of OASIs in RCTs. As recognized in one of the described trials, it appears to be difficult for obstetric care providers to adhere to the study protocol, as many of the trials included show only a small difference in the episiotomy rate between the two study arms. Only in the two oldest studies, dating from the 1980s, was the selective use of episiotomy really selective, with rates of 7–10%. (Evidence level 1+).

More recently, the EPITRIAL from Israel was published, including 676 primiparous women, in which “no episiotomy” was compared with “standard care,” with OASIs as the primary outcome [246]. OASI rates in women with SVD were 2.1% and 3.1% respectively. Episiotomy rates in both groups were 18.6% and 31.6% respectively (Evidence level 1++).

All these trials show that a more restricted approach toward the use of episiotomy may be possible without an increased risk of OASIs. The problem is that in these trials, the episiotomy rate in the arms with selective use showed a wide range, which makes these trials incomparable. Furthermore, these trials give no guidance on the optimal rate of episiotomy for the prevention of OASIs in SVDs.

Evidence from large observational studies based on a well-established database may be useful to identify risk-modifying factors for OASIs, e.g., MLE/LE. Older studies based on national databases have shown conflicting results. A large study from Norway based on data from 1967 to 2004 showed no protective effect of MLE in nulliparous women with SVDs (OR 1.0; 95% CI 0.97–1.1), whereas the OASI risk was increased in parous women (OR 1.3; 95% CI 1.2–1.5) [247]. A similar study from Finland containing data from more than 500,000 births between 1997 and 2007 showed a significantly reduced rate of OASIs with LE in nulliparous women with SVD (OR 0.83; 95% CI 0.75–0.92), with a risk-increasing effect in multiparous women, although the exact calculation of this risk in SVDs was not given [248] (Evidence level 1−).

In a large population-based study from the UK describing a cohort of more than 1 million women, MLE was significantly associated with a lower risk of OASIs in SVDs (aOR 0.57; 95% CI 0.51–0.63) [1] (Evidence level 2+).

A recently published Dutch population-based study containing over 200,000 nulliparous women also showed a significantly lower rate of OASIs in SVDs with the use of MLE (aOR 0.3; 95% CI 0.30–0.34), but additionally the importance of the accumulation of risk factors of OASIs [26]. In this study, it was shown that the number needed to treat (NNT) for an MLE in general to prevent one OASI was 31. With accumulation of risk factors such as high fetal birthweight and prolonged second stage of labor, this number was only 9 in the situation of a SVD in a nulliparous woman delivering a baby weighing more than 4000 g after a second stage of more than 120 min (Evidence level 2+).

Considering the use of MLE/LE in the prevention of OASIs, one needs to consider the possible drawbacks of these types of episiotomy. In general, use of MLE/LE is associated with more blood loss, pain immediately after birth, and possibly dyspareunia. The latter two may be mitigated with the best possible technique of repair, but it is doubtful whether an optimal technique of repair would prevent these sequelae.

With all the evidence on risk factors it is still not possible to counsel an individual woman about her real-time risk of OASIs during the birth of her baby. It would be very helpful if properly designed prediction models become available, based on robust representative data with sufficient numbers of OASIs. In the era of shared decision-making, women deserve optimal counseling about their risks and the possible interventions to mitigate these risks. Counseling is still hampered because of the lack of well-validated models.

#### Episiotomy in Operative Vaginal Births

The risk of OASIs is, compared with SVD, known to be significantly higher in OVBs. Nevertheless, the prevailing World Health Organization (WHO) guideline dating from 2018 “Intrapartum care for a positive childbirth experience” states that the role of episiotomy in obstetric emergencies such as fetal distress requiring OVBs remains to be established, whereas the role of episiotomy in other indications for OVB was not addressed at all [249]. The most recent RCOG GTG guideline, no.26, Assisted Vaginal Birth, concludes that there is no robust evidence to support the routine or restrictive use of episiotomy during OVB. As both guidelines have a great impact on daily obstetric practice around the globe, these conclusions may have profound consequences for women delivering with vacuum or forceps assistance.

Both guidelines are almost entirely based on the results of RCTs. As stated earlier, this may lead to a skewed view on the possible role of MLE or LE during OVB.

The recommendations in the GTG are primarily based on the pilot RCT by Murphy et al., published in 2008, in which the restrictive use of MLE was compared with liberal use [234]. A total of 200 women were randomized, of whom 175 actually delivered with forceps or vacuum assistance. For the analysis of the primary outcome of OASIs, both types of OVB were analyzed together. However, the absolute number of OASIs in this pilot RCT was very low, resulting in only very limited statistical power. Furthermore, the rate of MLE in the restrictive arm of the trial varied enormously: 17% in women delivered with vacuum assistance versus 64% in women delivered with forceps assistance. This makes it questionable whether it is correct to combine the results of the births with both instruments (Evidence level 1−).

Recently, an RCT from Sweden on this subject was published by Bergendahl et al. [250]. In this trial 702 nulliparous women were randomized between a standardized LE at 60° starting 1–3 cm from the posterior fourchette and no episiotomy unless considered indispensable. The OASI rate with episiotomy was 6% vs 13% in women delivered without episiotomy which resulted in a risk ratio of 0.47 (96% CI 0.23–0.78). No significant differences were noted between groups with regard to postpartum pain, blood loss, neonatal outcomes, or total adverse events, but the intervention group had more wound infections and dehiscence (Evidence level 1−).

In 2022, a large systematic review and meta-analysis on the association of MLE and LE with OASIs was published, describing the analysis of 31 RCTs and non-RCTs including a total of more than 700,000 births [251]. This meta-analysis showed a significant risk reduction with MLE/LE during OVB in nulliparous women with an OR of 0.51 (95% CI 0.42–0.84) in vacuum-assisted births and an OR of 0.32 (95% CI 0.29–0.61) in forceps-assisted births. Although the OASI rate in multiparous women with both types of OVB was lower when MLE/LE was applied, this association did not reach significance in either vacuum extractions or forceps-assisted births. As most studies were observational, heterogeneity was significant, and the risk of bias should be considered (Evidence level 1+).

An earlier review paper from 2019 described the association of MLE/LE with OASIs in OVBs on the basis of the results of five observational studies using large population-based registries [252]. In all five studies the use of MLE/LE during OVB was associated with a significantly lower risk of OASIs in nulliparous women. Three studies addressed this effect in multiparous women. Two Dutch studies showed a protective effect with MLE in multiparous women, whereas one study found no significant effect of LE. Interestingly, this study described that risk association of MLE/LE with OASIs in OVB may depend on the actual episiotomy rate. One of the described studies from Denmark showed a less pronounced effect, with an episiotomy rate of only 28.7% of vacuum extractions compared with a MLE rate of more than 70% of vacuum extractions in the studies from the UK and the Netherlands, in which the effect of MLE was much stronger. This phenomenon was described earlier in a review by Lund et al. [253] that described 15 observational studies. In studies with an episiotomy frequency of >75% (5 studies, 53,435 women) the pooled OR of OASIs was 0.37 (95% CI 0.15–0.92) compared with a pooled OR of 0.67 (95% CI 0.45–1.01) in the studies with a frequency of episiotomy <75%. This phenomenon demonstrates the struggle of clinicians to identify which women may benefit from a MLE/LE (Evidence level 2++).

The difficulty in identifying women at a high risk of OASIs demonstrates the need for adequate prediction models in both SVD and OVB. These models should have the ability to counsel women with both.

#### Episiotomy Angle

Use of an episiotomy to reduce the tensile forces of the perineum during crowning of the fetal head or breech during birth is one of the most common procedures in daily obstetrics. The rate of use has declined in the most recent decades as stated earlier. One of the possible reasons for this decline was the debate about the protective potential of this procedure.

In 2012 the different types of episiotomy known from medical literature and textbooks were described and a standardization of the characteristics of each type of episiotomy was proposed [243]. As described, the most common type of episiotomy was the MLE, which was defined as an incision starting within 3 mm of the midline in the posterior fourchette, laterally directed at an angle of at least 60° from the midline toward the ischial tuberosity. This description was based on work that showed that to achieve an angle of 45°, supposed to be “the ideal angle,” an incision angle during crowning of at least 60° is required [254].

The importance of the angle of the MLE was demonstrated in two studies from 2006, in which the risk of OASIs increased with a more acute angle of the episiotomy [255, 256]. One of these studies showed that the risk of OASIs decreased relatively by 50% with every 6° increase in the angle of the episiotomy [255] (Evidence level 2−).

A case–control study from Norway showed that this effect disappeared when the scar angle of the episiotomy exceeded 60°, demonstrating that there is a maximum angle of episiotomy to achieve a reduction in the risk of OASIs [257] (Evidence level 2−).

More recently, special devices have been developed to ensure a proper angle of episiotomy at the moment of incision when an MLE is used. Two reviews with regard to the use of the Episcissors-60™ showed that with this type of scissors, an adequately angulated MLE was achieved more often and was associated with a lower rate of OASI than episiotomies cut with standard scissors [258, 259] (Evidence level 1−).

Recently, a small RCT was published studying the use of the “Episiometer,” a drawing showing the proper angle of episiotomy ready for clinical use during birth, and conventional “eyeballing episiotomy” [260]. With the use of the new device the OASI rate was 0.6%, compared with 4.9% with the conventional method of episiotomy (adjusted relative risk [aRR] 5.9; 95% CI 0.75–46.1). Significantly more women had an adequately angulated episiotomy with the use of the new device (Evidence level 2++).

#### Vaginal Birth After Cesarean Section

The association between VBAC and OASIs is complex. However, a systematic review including 23 articles in 11 countries and a total of 3,980,918 women (241,468 women with VBAC) showed an overall higher OASI prevalence in the VBAC group (8.23%) than in primiparous women (6.42%), corresponding to a 1.27 odds of OASI with VBAC (95% CI 1.10–1.47) [261]. There were no differences in OVB rates between the groups. After exclusion of studies that may have included prior vaginal births in the VBAC group, the odds of OASI further increased with VBAC compared with primiparous women (1.35; 95% CI 1.14–1.59) (Evidence level 2−).

A single-site retrospective cohort study of 1411 women undergoing VBAC showed that a predicted low probability of VBAC correlated with a higher likelihood of OASIs than a predicted VBAC of >80% [262] (Evidence level 2+).

Another study evaluated risk factors for OASIs during a VBAC in a cohort of 1375 women. In this study, a higher risk of OASIs was associated with advanced maternal age, higher infant birthweight at the initial CS and the current VBAC, and an urgent indication for prior CS. In this cohort, MLE reduced the risk of OASIs by 50% [263]. A similar but larger study on 28,535 women with VBAC compared with 275,439 primiparous women found similar risk factors for OASIs, except that the indication for emergency CS was further evaluated. In this study, the aOR for OASI with VBAC after an emergency CS indicated for failure to progress in labor was 1.18 (95% CI 1.08–1.29). However, an emergency CS indication of suspected fetal distress was not associated with OASIs [264] (Evidence level 2−).

#### Perineal Body Length

Perineal length is dependent on the moment of measurement. The described average length of the perineum is 39 mm (range 37–41 mm) in the late antenatal period or the first stage of labor, as measured in studies on the subject of perineal length and the possible risk of perineal lacerations [86]. This length may increase by 50–60% at crowning to 60 mm.

Several observational studies have shown that a shorter perineal length is an independent risk factor for the occurrence of OASIs. In one study, where a cut-off value of 25 mm was used (measured during the first stage), the risk of OASIs was significantly higher with a shorter perineum (40% vs 5.6%) [265]. In a more recent prospective observational study from the USA this association was confirmed, with an increased risk of OASIs with a perineal length of less than 35 mm [266]. A study that used 3D EAUS 6 weeks after the first birth showed signs of OASI in 40% of women with a perineal length of <30 mm measured at 35–37 weeks versus 11% in those with a longer perineal length [267] (Evidence level 2+).

It is important to realize that 14–20% of women have been reported to have a perineal length of <30 mm and may be at a higher risk of sustaining an OASI during vaginal birth.

Sound evidence on preventive measures taking into account the factor of perineal length is lacking to date.

#### Female Genital Mutilation/Cutting

Female genital mutilation/cutting is a cultural practice that violates the human rights of girls and women. FGM-C involves partial or total removal of the external female genitalia and it can vary in severity. Type III, also called infibulation, is a severe type of FGM-C performed by cutting the labia minora and/or majora and forming a seal over the vestibule, thereby narrowing the birth orifice. This can be deinfibulated in the pre-conception, antenatal, or intrapartum timeframes to allow for childbirth.

A large systematic review and meta-analysis on the effects of female genital cutting on physical health outcomes showed a tendency toward an increased risk of perineal lacerations in general in the four studies addressing this issue (aOR 1.39; 95% CI 0.99–1.95) [268]. In a different meta-analysis from the same authors that contained more and older studies, a stronger association of FGM with perineal lacerations was reported (RR 1.38; 95% CI 1.07–1.79) [269] (Evidence level 2++).

A large population-based study from Sweden showed that women with FGM who delivered in Sweden had an almost tripled risk of OASI in comparison with women without FGM [270]. Although this differed slightly according to the country of origin, the risk remained significant in all sub-cohorts. This study confirmed an earlier study from Sweden where this association was described for women born in East Africa. In this study, women from Somalia sustained the highest risk (aOR 2.72; 95% CI 2.08–3.54), followed by women from the region of Eritrea–Ethiopia–Sudan (aOR 1.80; 95% CI: 1.41–2.32) when compared with women born in Sweden. Both Eastern African regions are known for a high rate of FGM-C. No association with the occurrence of OASIs was found in women born in other regions of Africa. In both studies, the extent of FGM was unknown owing to the nature of the two studies, using a national registry system in which the type of FGM was not recorded (Evidence level 2−).

A historical cohort study of Somali-born women using a Medical Birth Registry in Norway also showed a significantly higher incidence of OASIs among all types of FGM-C than in the general Norwegian population (10.3% vs 5%), but there were no differences between FGM-C types (10.2% Type I–II, 11.3% Type III, 15.2% unclassified; *p* = 0.17) [271] (Evidence level 2+).

Although studies from the African continent itself are scarce and difficult to trace, an older study from Ethiopia reported a higher rate of third-degree tears amongst nulliparous women after FGM than among women without FGM [272] (Evidence level 2−).

In the population-based study from Sweden no direct calculations were made regarding interventions that might lower the risk of OASIs for women after FGM-C. However, the increasing OR for FGM-C after adjusting for episiotomy suggests a protective effect of episiotomy in these women [270]. In the Norwegian cohort study, intrapartum deinfibulation was associated with a lower incidence of OASIs than pre-labor deinfibulation. However, in a meta-analysis that included only two small studies and a total of 59 participants, there was no difference in perineal lacerations or need for episiotomy based on the timing of deinfibulation. There was, however, a trend toward fewer perineal lacerations in those who had intrapartum deinfibulation than in those who were not deinfibulated (OR 0.27; 95% CI 0.06–1.16) [273]. In general, deinfibulation is strongly recommended by the WHO to reduce the risks of obstetric complications, including the potential to reduce perineal lacerations [274]. More recently, a prospective cohort compared intrapartum outcomes between 174 women with Type III FGM-C undergoing intrapartum deinfibulation with 171 women with no or Type I–II FGM-C. The MLE rate was higher with Type III FGM-C. In those who underwent deinfibulation, MLE was associated with a lower incidence of perineal lacerations, including OASIs [275] (Evidence level 2−).

## Section 6: Management of Subsequent Pregnancies

**Clinicians should offer individualized counseling to all women with a history of OASI, using a collaborative, patient-centered approach to empower informed decision-making regarding the mode of subsequent birth. Both the counseling discussion and the patient’s preferred birth route should be clearly documented (B).**

All women with a previous OASI require individualized counseling regarding the mode of subsequent birth, utilizing a collaborative and patient-centered approach. During counseling, it is essential to provide comprehensive information regarding risks associated with each mode of birth to support informed decision-making. Women with a history of OASI commonly identify two primary concerns with regard to future birth: the risk of rOASI and the potential for development of de novo or worsening of anorectal symptoms [276]. Women should be counseled that although subsequent vaginal birth is associated with an elevated risk of rOASI, elective CS may not provide protection against development of anorectal symptoms [277]. Shared decision-making is essential, with 34.9% of women reporting that the counseling they received significantly influenced their choice of birth route [278]. This discussion, along with the patient’s preferred mode of birth, should be thoroughly documented (Evidence level 1−).

### What Is the Risk of Recurrent Obstetric Anal Sphincter Injury in a Subsequent Birth?

**Women should be advised that the overall risk of recurrent OASI is 6.1% (B).**

Women who experience OASI are at a fivefold (OR 4.9) increased risk of rOASI in subsequent vaginal birth compared with multiparous women without a prior OASI [277, 279]. The risk of OASI in subsequent vaginal birth ranges from 2.0% to 13.4%, with an overall risk of recurrence of 6.1–6.6%, whereas it is approximately 1.08–1.5% in multiparous women without a previous OASI [277, 280, 281]. The number needed to harm is 20 (one in 20 women with a history of OASI undergoing vaginal birth will likely experience rOASI) [277]. Among women who experience two OASIs, the risk of recurrence remains elevated in a subsequent vaginal birth, with a reported incidence of 3.1% [280] (Evidence level 1−).

Women with a history of OASI can be reassured that they will likely not experience a recurrence. The most frequently reported perineal laceration following OASI is a second-degree perineal laceration, reported in 59.4% of vaginal births [282]. For women who have a vaginal birth following previous OASI, a thorough postpartum perineal examination is essential to identify the degree of perineal trauma. If rOASI is diagnosed, repair and postpartum care should follow the recommendations detailed in this guideline (Evidence level 2+).

### What are the risk factors and protective factors for rOASI?

**Clinicians should be aware that most risk factors associated with rOASI are nonmodifiable or only become evident during labor (C).**

**Selective use of MLE at the time of subsequent vaginal birth, when clinically indicated, may offer a protective effect and reduce the risk of rOASI (C).**

The risk factors for recurrence, as outlined in the below table, are important considerations in counseling for subsequent birth planning.

**Table:** 

Maternal	Fetal	Obstetric
Maternal age (years) [282–284]	Fetal macrosomia (birth weight >4 kg) at subsequent birth (OR 2.1) [280, 282, 285]	Duration of the second stage ≥30 min (OR 1.8) [285]
Maternal age ≥40 years at subsequent birth (OR 1.34–1.95)[280]	Occiput posterior position at subsequent birth (OR 1.41–2.85) [280, 283]	Augmentation with oxytocin (OR 1.48) [283]
History of 2 previous OASIs (OR 10.6) [280]	Higher gestational age [283]	OVB at subsequent birth (OR 3.46) [64]
Wound infection at the index birth complicated by OASI (OR 9.97)[286]		Shoulder dystocia at subsequent birth (OR 1.98–4.27) [280, 283, 284]

*OASI* obstetric anal sphincter injury, *OR* odds ratio, *OVB* operative vaginal birth

Concerning protective strategies, a systematic review and meta-analysis encompassing 11 studies concluded that episiotomy at the time of subsequent birth does not appear to be either a definitive risk factor or inherently protective against rOASI [283]. A notable limitation of this review was the lack of differentiation in many of the studies included between episiotomy techniques, specifically midline versus MLE. In contrast, recent research focusing specifically on MLE found a potential protective benefit. In a multi-site retrospective population-based study of four NHS trusts including 2272 women, D’Souza et al. reported an approximately 80% reduction (OR 0.21; 95% CI 0.080–0.524)) in the risk of rOASI among women who received a MLE during subsequent vaginal birth. Notably, their study demonstrated a low NNT, with 8 MLEs required to prevent 1 rOASI across all modes of vaginal birth, and 10 in the context of spontaneous vaginal birth [282]. These findings support the selective use of MLE, when clinically indicated, as a potentially effective intervention to reduce the risk of recurrence. Further research is required to evaluate the effectiveness of intrapartum strategies in the prevention of rOASI (Evidence level 1−).

### What Is the Risk of Development of Anorectal Symptoms After Subsequent Birth Post-Obstetric Anal Sphincter Injury?

**Future research is required regarding the impact of mode of subsequent birth on the development or progression of anorectal symptoms in women with a history of OASI (B).**

**Elective CS may not be completely protective against prevention of de novo or worsening short-term and long-term anorectal symptoms in women with prior OASI (C).**

**Recurrent OASI is associated with an increased risk of de novo anorectal symptoms and with greater symptom severity (B).**

The presence of index OASI predisposes women to the development of anorectal symptoms, including fecal urgency, fecal incontinence, and flatal incontinence [287]. The presence of even mild anorectal symptoms can significantly impair quality of life [288].

The existing literature on the impact of subsequent birth on the development of anorectal symptoms is inconclusive, with some studies reporting the onset or worsening of anorectal symptoms following birth, whereas others demonstrate no significant change in symptomatology irrespective of the mode of subsequent birth [281, 282, 289, 290]. A recent systematic review and meta-analysis of ten studies including 970 participants revealed no difference in anal incontinence in women with and in those without subsequent birth post-OASI [281]. For women who have a subsequent birth, the presence of rOASI is associated with an increased risk and a cumulative burden on the severity of anorectal symptoms (OR 1.6) [277, 279, 291]. Anal incontinence was reported in 26.2–50% of women following two OASIs, compared with 14.3–37.9% of women with a single OASI [2, 291–293]. Therefore, prevention of rOASI is important during subsequent births.

Some women with a prior OASI may request an elective CS [279]. Although CS prevents rOASI, it is important to acknowledge that anorectal symptoms are influenced by multiple factors, such as weakness in the pelvic floor musculature, and CS may not necessarily confer protection against the development of de novo or worsening anorectal symptoms in women with a history of OASI [294–296]. In two recent systematic reviews and meta-analyses, there was no association between mode of subsequent birth and the risk of de novo or worsening anal incontinence [277, 281]. There was no difference in short-term anal incontinence at 3–24 months postpartum and long-term anorectal symptoms ≥5 years postpartum after subsequent birth [281]. There was also no difference in the development of anal incontinence in asymptomatic women irrespective of mode of birth, but this was based on a smaller sample size (two studies that included 220 participants) [281]. New onset or worsening of anal incontinence ranged from 3.6% to 23.5% following subsequent birth and from 0% to 21.3% following elective CS [2, 197, 297–299]. An RCT by Abramowitz et al. assessed the effect of elective CS versus vaginal birth in 222 asymptomatic women with ultrasonographic evidence of prior OASI, reporting no significant difference in anal incontinence between the two birth modes at 6–8 weeks and 6 months postpartum [299].

However, the available evidence is limited by methodological constraints, including small sample sizes, short-term follow-up, heterogeneity of studies, and none meet all the quality criteria [277, 281, 290]. Currently, available evidence regarding the impact of mode of birth on long-term anorectal symptoms originates from one observational study consisting of 1978 participants [2, 281]. Observational studies may be subject to bias owing to potential confounding variables [277]. Additionally, the findings from the single RCT [299] may have limited generalizability owing to several factors, and these have been highlighted by Okeahialam et al. [300]:

1. Concerns regarding the definition of a significant anal sphincter defect on EAUS utilized in the inclusion criteria.
2. Most of the participants (91.0%) had OVB with forceps, 9.0% had SVB, whereas no women had a vacuum-assisted birth.
3. The majority of women randomized had no perineal laceration (71.1%).
4. Only 14.7% of women randomized had a third-degree perineal laceration at the index birth and no women had a fourth-degree perineal laceration.
5. The anal sphincter complex function was not assessed with ARM.
6. The timeframe for development of anorectal symptoms (6–8 weeks and 6 months postpartum) was relatively short.
7. The absence of subgroup analysis to inform recommendations based on the size of the residual anal sphincter defect observed on EAUS.

Consequently, the conclusions of this RCT in terms of outcome after acute OASIs are questionable, which the authors acknowledge: “It remains to be shown whether our findings can be replicated in different populations including only obstetric anal sphincter injuries that are diagnosed at birth” [299].

It is important to note that the prevalence of anorectal symptoms is typically higher in the puerperal period and decreases as the pelvic floor recovers postpartum [301, 302]. However, these symptoms may become more prevalent over time as the pelvic floor and anal sphincter function decline with age [301, 302]. Consequently, anorectal symptoms may develop years after the index OASI [303]. Long-term anal incontinence has been reported in approximately half (38.9–64.5%) of women after subsequent birth post-OASI [4, 257, 281, 304]. Fecal incontinence has been reported in 36.8% of women aged over 40 years who have a history of OASI [281]. The presence of transient or permanent anorectal symptoms following index birth complicated by OASI is linked to the development of long-term anorectal symptoms irrespective of mode of subsequent birth [281].

Owing to low-quality evidence, it is difficult to create clinically meaningful conclusions [281]. Further high-quality research is required to more accurately determine the effect of birth route on anorectal symptoms postpartum after subsequent birth post-OASI and long-term to guide individualized decision-making (Evidence level 2−).

**How can women with OASIs be counseled regarding the mode of subsequent birth?**

**Advanced: Clinicians should be aware that there are published protocols to guide the mode of birth counseling in a subsequent pregnancy, which include symptom assessment, EAUS, and ARM (B).**

**Desirable: In the absence of EAUS and ARM, symptoms,rectal examination findings, and a history of a fourth-degree tear can be used to guide the mode of birth counseling (C).**

1. Assessment of sequelae: Review the physical and emotional consequences from prior OASI and their impact on the woman’s overall quality of life.
2. Clinical evaluation: Discuss findings from physical examination and relevant investigations to assess anal sphincter integrity and function (EAUS, ARM). Notably, between 75% and 95% of women agree to deliver according to recommendations made by their health care provider based on EAUS and ARM findings [197, 276].
3. Family planning: Explore the woman’s future reproduction plans and her desire for additional pregnancies, as this may influence the risk−benefit assessment regarding the mode of subsequent birth.
4. Risks and benefits of each birth route: Provide a balanced evidence-based discussion on the risks and benefits associated with vaginal birth, elective CS, and intrapartum CS. This should be tailored to the individual’s clinical context, carefully weighing whether the potential morbidity associated with CS is justified by the possible benefit of preventing further injury to the anal sphincter complex.
5. Risk of rOASI: Explain the risk of recurrence, incorporating the women’s individualized risk factors, and discuss potential strategies to reduce the risk, such as the selective use of MLE when indicated.
6. Risk of de novo or worsening anorectal symptoms: Present the potential for new-onset or worsening of anorectal symptoms with each mode of birth.
7. History of pelvic surgery: Consider the implications of any prior pelvic floor surgeries and interventions (secondary anal sphincter repair, SNM) and their impacts on the mode of birth.
8. Patient and family preferences: Integrate the woman’s values, preferences, and concerns, along with those in her support system, into the shared decision-making process, ensuring that she feels well-informed, supported, and that her choice is respected.

**Fig. 3:**
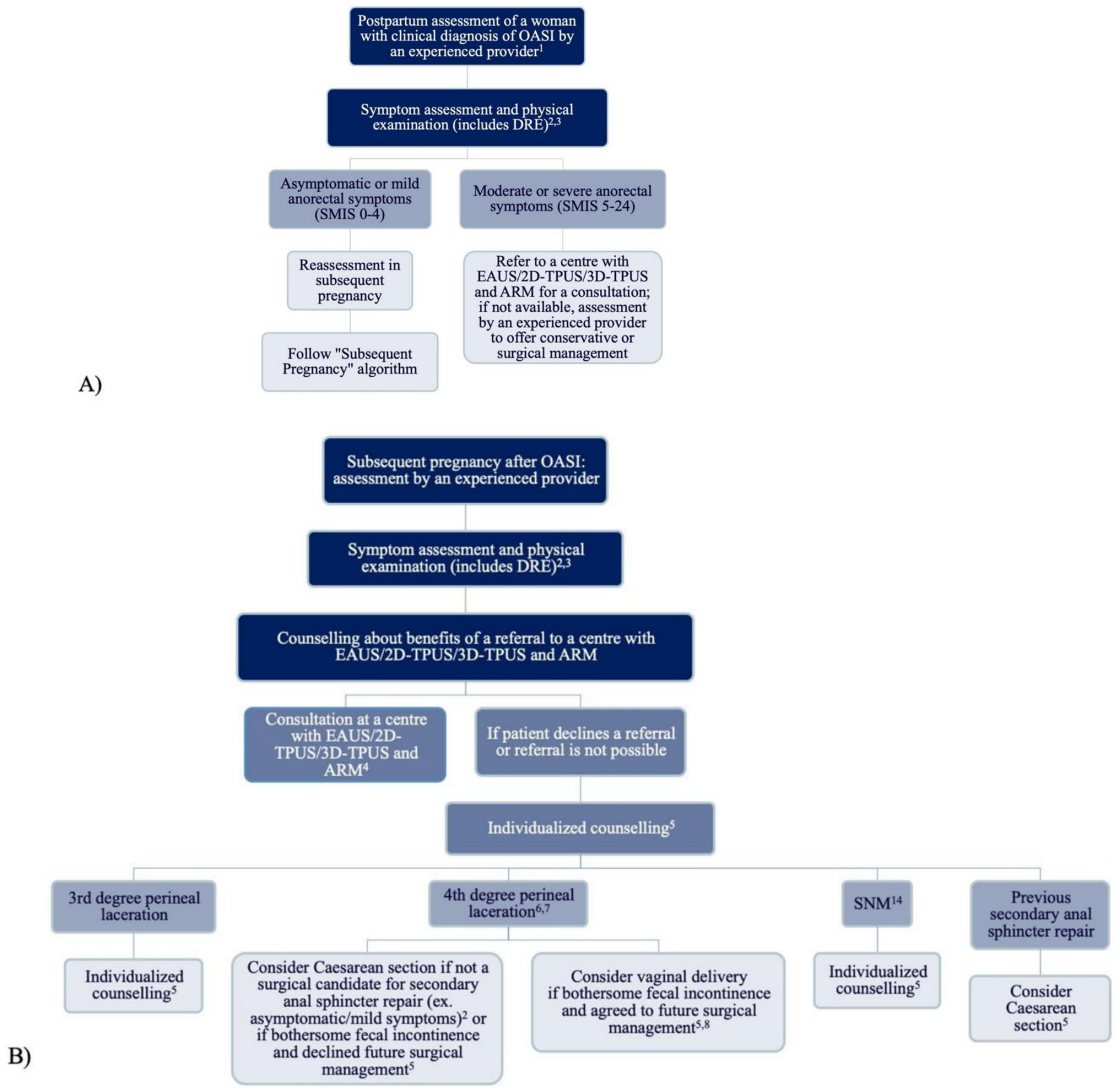

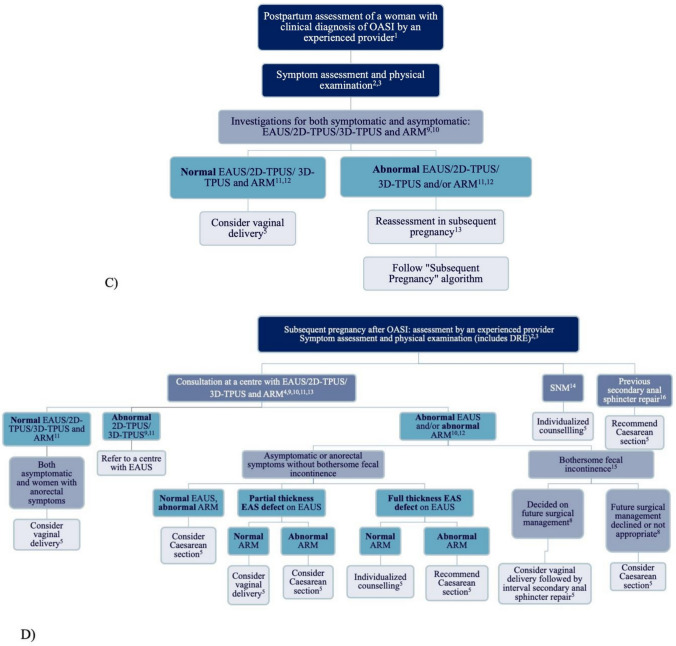
Recommendations regarding postpartum management and subsequent pregnancy after obstetric anal sphincter injury (OASI) [305]. **A** Recommendations regarding postpartum management for women with OASI for centers without access to endoanal ultrasound (EAUS)/two-dimensional transperineal ultrasound (2D-TPUS)/three-dimensional transperineal ultrasound (3D-TPUS) and anorectal manometry (ARM). **B** Recommendations regarding management of subsequent pregnancy for women with a history of OASI for centers without access to EAUS/2D-TPUS/3D-TPUS and ARM. **C** Recommendations regarding postpartum management for women with OASI for centers with EAUS/2D-TPUS/3D-TPUS and ARM. **D** Recommendations regarding management of subsequent pregnancy for women with a history of OASI for centers with EAUS/2D-TPUS/3D-TPUS and ARM. Algorithms created by Maria Giroux, MD FRCSC (Regina, SK, Canada) and Abdul Sultan, MBChB, MD, FRCOG (Croydon, Surrey, UK). ^1^Assessment is typically performed at 6–8 weeks postpartum [306]. If available, women with a clinical diagnosis of OASI are referred to a dedicated clinic. Women requiring postpartum surgical revision (e.g., wound dehiscence) who are found to have missed OASI and undergo anal sphincter repair should be followed using the same algorithms as women with a clinical diagnosis of OASI at the time of delivery. The timing of postpartum assessment varies among dedicated clinics [173]. EAUS and ARM are performed at least 6 weeks postpartum. ^2^Anorectal symptoms include fecal urgency, fecal incontinence, and flatal incontinence. St. Mark’s Incontinence Score (SMIS) is used for assessment of anorectal symptoms [287]. Severity of anorectal symptoms is classified as mild (0–4), moderate (5–8), and severe (9–24). ^3^Digital Rectal Examination Scoring System score may be used for digital rectal examination (DRE) [307]. ^4^During the third trimester (after 28 weeks) based on expert opinion. This timeline may be tailored toward an individual’s unique circumstances. ^5^Counseling to support shared decision-making regarding subsequent mode of delivery using a collaborative individualized approach considering the following components: Current sequelae and impact on quality of life; clinical assessment and investigations; reproductive goals and future childbearing; risks and benefits of each delivery option; risk of recurrent obstetric anal sphincter injury (rOASI); risk of de novo or worsening anorectal symptoms; history of pelvic floor surgery or interventions; patient and family values and preferences. This guideline does not endorse routine elective Caesarean section following OASI, but rather one of the options to be considered within individualized counseling. ^6^Only 7% of women with a fourth-degree perineal laceration are asymptomatic with normal EAUS and ARM findings and 82–86.6% have abnormal EAUS relevant to counseling [308]. ^7^Women with no or mild anorectal symptoms with a traumatic cloacal defect may be offered an elective Cesarean section owing to the possibility of tissue breakdown with a subsequent vaginal delivery based on expert opinion [309]. ^8^Surgical management for anorectal symptoms options may include secondary anal sphincter repair, sacral neuromodulation (SNM), artificial anal sphincter, or gracilis neo-anal sphincter reconstruction after recovery postpartum and/or after completion of childbearing. ^9^If EAUS is not available, 2D-TPUS/3D-TPUS can be utilized as a screening tool for the presence of residual anal sphincter defect. Women with no evidence of a residual anal sphincter defect on 2D-TPUS/3D-TPUS do not require a referral for EAUS [177]. Women with an anal sphincter defect identified on 2D-TPUS/3D-TPUS should be referred to a center with EAUS for confirmation of the diagnosis [177]. ^10^If ARM is not immediately available, then ARM may be deferred and performed during a subsequent pregnancy or prior to surgical management of fecal incontinence. In centers without access to ARM, assessment of the anal sphincter function may be performed using DRE in combination with EAUS, 2D-TPUS, or 3D-TPUS, with the understanding that these approaches have not been formally validated against ARM [179, 181]. ^11^Normal EAUS/2D-TPUS/3D-TPUS is defined as normal continuity of the anal sphincter complex (no evidence of previous OASI) or scar in the EAS [127, 177]. Abnormal EAUS/2D-TPUS/3D-TPUS is defined a residual anal sphincter defect in the EAS [177, 276]. The presence of an IAS defect does not impact counseling regarding subsequent mode of delivery [276]. ^12^Normal ARM is defined as incremental maximum squeeze pressure (iMSP ≥20 mmHg [197]. Abnormal ARM is defined as iMSP <20 mmHg [197]. ^13^Women do not require repeat EAUS in subsequent pregnancy unless there is uncertainty regarding original ultrasound findings [197]. For women with abnormal ARM postpartum, repeat ARM during subsequent pregnancy is recommended [310, 311]. ^14^The optimal mode of delivery after SNM is unknown and an individualized approach is required. ^15^Provider to assess appropriateness for surgical management, such as secondary anal sphincter repair, based on the presence and size of the anal sphincter defect, contractility of the residual anal sphincter muscle, and the surgical skillset of the operator. Clinicians should consider other potential etiologies or contributors to anorectal symptoms (e.g., irritable bowel syndrome), which may not improve with surgical management. If there is a significant residual anal sphincter defect with bothersome fecal incontinence and anal sphincter contractility on DRE, then consider secondary anal sphincter repair. If there is no anal sphincter contractility on DRE, then refer for pelvic health physiotherapy and/or SNM. ^16^Women with clinical diagnosis of OASI at the time of delivery undergoing secondary anal sphincter repair are recommended to have a Cesarean section for subsequent pregnancies

Figure 3 presents algorithms derived from the Canadian 2025 guideline outlining recommendations for the mode of subsequent birth in women with a history of OASI, tailored for centers with and those without access to investigative tools. These algorithms were developed based on expert consensus and incorporate the best available evidence to date [2, 277, 279, 281, 282, 289–299]. However, they should be interpreted with the understanding that future long-term research is necessary to establish the optimal mode of subsequent birth in this patient population. If vaginal birth is selected, birth should be managed by an experienced clinician, with an individualized threshold for intrapartum CS based on the presence of risk factors for rOASI (Evidence level 2−).

### Do Women Regret Their Mode of Subsequent Birth?

**Clinicians should be aware that the majority of women with prior OASI are satisfied with the mode of subsequent birth (C).**

Currently, evidence of regret related to the mode of subsequent birth post-OASI is limited. A small study involving 50 participants reported that 90% of women experienced no or mild regret regarding their decision on the mode of subsequent birth [278] (Evidence level 2−).

A recent systematic review reported no difference in levels of satisfaction and regret based on the mode of birth [281]. Further research is needed to assess long-term regret and satisfaction in this patient population (Evidence level 1−).

## Section 7: Medicolegal, Education, and Resources

### Governance, Risk, and Litigation

Litigation related to OASIs is on the rise in jurisdictions that have assessed it. The majority of cases are related to the use of an episiotomy and failure to identify the injury following birth, leading to subsequent anal incontinence, rectovaginal, or anovaginal fistulae [81, 312].

England saw 441 claims from 1 April 2000 to 31 March 2010, in which allegations of negligence were made owing to obstetric perineal trauma [312]. This was the fourth highest number of claims in obstetrics. The total value of those claims, including both damages and legal costs, was estimated to be $US 40 million (£31.2 million) or a mean of $US 90,000/case. The majority (85%) of these claims were related to misdiagnosis of perineal trauma [312]. A similar study in the USA reported that of the one third of cases that found in favor of the plaintiff, all involved episiotomy, and plaintiff awards ranged from $US 110,000 to $US 841,810 [81].

The occurrence of an OASI currently falls within the standard of care in most jurisdictions, as it is considered a known complication of vaginal birth. Successful litigation seems to primarily revolve around the use of an episiotomy, failure to recognize the anal sphincter damage after birth, and an inadequate repair, including poor technique, poor selection of materials, or poor healing [81, 313].

Maternity units can minimize the risk of litigation related to OASIs through the development of a clear protocol for the prevention and management of OASIs. This should include documentation of the anatomical structures involved (Section “Background and Epidemiology”). Proven methods to prevent OASIs (Section 5: Risk Factors, Prediction, and Prevention) and the site’s preferred method of repair, including suture materials to use (Section “Repair Techniques”). The site protocol should mandate proper informed consent that accurately describes risks of adverse outcomes for both vaginal birth and repair of OASIs (see Section “Consent”) This standard is seemingly not currently met in many jurisdictions [314]. The ubiquitous presence of episiotomy in US litigation related to OASIs has led some authors to advocate for avoiding the use of episiotomy, although the use of episiotomy with OVB has been shown to decrease the incidence of OASIs [315]. At a minimum, the use of midline episiotomy should be avoided [81]. Site standards should also mandate thorough and specific documentation in the medical record, comprehensive discharge instructions, and counseling (Section “Patient Education and Counseling”), and timely evaluation and referral to a specialist in OASI repair and management who has received specific training in the evidence-based repair of OASIs. If these standards are followed, providers facing OASI-related litigation are more likely to prevail [81].

### Provider Education and Training and Competency Tools

**Health care providers involved in vaginal births must undergo mandatory OASI education and simulation training (GPP).**

**Standardized training curriculum focusing on OASI prevention, diagnosis, and repair techniques should be developed and implemented (GPP).**

**Promote interdisciplinary training sessions to foster teamwork and improve communication during labor and birth (GPP).**

**Provide ongoing training opportunities, including updates on new evidence and practices (GPP).**

Effective provider education and competency training are essential to minimize the incidence of OASIs and enhance the quality of care provided to affected women. Key components include:

1. Knowledge of OASI prevention, diagnosis, and management.
2. Standardized training to improve clinical skills related to OASI care.
3. Assessment tools to ensure competency in managing OASIs.
4. Adherence to evidence-based practices to reduce OASI-related morbidity.

#### Provider Education

##### Importance of Education

Education is foundational in equipping obstetricians, midwives, family physicians [316], and other health care providers with the knowledge required to identify risk factors, implement preventive measures, and provide appropriate care for women with OASIs.

Educational interventions can significantly reduce the incidence of OASIs and improve maternal outcomes [184]. Policy makers should therefore prioritize this area of maternity care with training programs and quality control, which should be implemented in high- as well as low-resource countries [79].

##### Core Content Areas

Provider education should cover the following core topics:

- Anatomy and pathophysiology
- Risk factors: Training should emphasize modifiable and nonmodifiable risk factors.
- Prevention strategies:
  1. The OASI care bundle (antenatal information to women, MPP, and MLE when indicated) has the potential for reducing perineal trauma during childbirth [11].
  2. The Stop Traumatic OASI Morbidity Project quality improvement project aimed at reducing OASI incidence by promoting the slowing down of the birth of the vertex and shoulders by encouraging women to stop pushing during crowning, by applying one hand to manually control birth speed, and by encouraging birthing positions other than the semirecumbent position, particularly the upright position [317].
  3. Evidence-based techniques, such as perineal protection during birth [318] and judicious use of MLE should be included in training modules.
  4. Continuous verification of the application and repetitive training are necessary [319].
- Diagnosis: Providers should learn systematic approaches for post-birth perineal examination to diagnose OASIs accurately. Underdiagnosis has been linked to poorer long-term outcomes.
- Repair techniques: Comprehensive knowledge of primary and secondary repair techniques, including end-to-end and overlapping suturing methods, is critical for effective management.

##### Training Methods

- Didactic lectures
- Online modules with interactive content provide distant learning and assist in acquisition of skills
  o http://www.perineum.net
  o RCOG OASI Care Bundle
- Workshops and seminars
  o Hands-on workshops on OASIs repair [214] have become an essential component of training over the past two decades, and in many obstetric curricula, a mandatory element of training portfolio. The hands-on component utilizes latex models and the repair of fresh animal anal sphincters.
  o Train the trainers program assists with the dissemination of skills (https://www.iuga.org/education/protect/about-protect)
- Case-based discussions [320]

#### Training Frameworks

##### Hands-On Simulation Training

Simulation-based training is an integral component of OASI education. Hands-on practice improves provider competency in perineal repair. Residents’ confidence level has been shown to increase with a greater number of simulations attended. Simulations, in addition to lectures, enhance residents’ knowledge and skills in OASI repair [321].

Training sessions may include:

- Anatomical models: High-fidelity models, including animal models, allow providers to practice identifying and repairing third- and fourth-degree tears.
- Step-by-step guidance: Facilitators should provide real-time feedback on suture placement, knot security, and tissue handling.
- Multidisciplinary approach: Collaborative training sessions with obstetricians, midwives, and nurses foster teamwork and standardized practices.

##### Prevention Techniques During Labor

Training should emphasize evidence-based practices for OASI prevention:

- Controlled birth of the fetal head using hands-on perineal support techniques [322, 323].
- Appropriate use of episiotomy, with preference for mediolateral over midline episiotomy in high-risk births. Standardization of episiotomy angle using Episcissors and associated training programs is aimed at optimizing the technique and promoting safe practice [324]. A quality improvement study showed that use of Episcissors-60 was associated with a statistically significant decrease in total OASI rate, but no difference in OASI rate within the subgroup that received an episiotomy [325]. More recently, a meta-analysis evaluating the incidence of OASIs before and after the implementation of this device in six observational studies (14,027 women) demonstrated a 2% risk difference (RD 0.02; 95% CI 0.03–0.00) in OASI incidence [258].

##### Repair Techniques

Providers should be trained in advanced suturing techniques for OASI repair.

##### Postpartum Care

Training must address postpartum follow-up care, including:

- Assessment of perineal healing, bowel function, and care.
- Guidance on pelvic floor exercises to support recovery.
- Referral pathways for women experiencing complications such as fecal incontinence or chronic pain.

#### Competency Tools

##### Competency Assessment Framework

Competency should be evaluated using structured assessment tools, ensuring that providers meet predefined standards. The framework should include:

- Knowledge assessment focusing on OASI prevention, diagnosis, and management.
- Skills assessment of suturing and repair techniques using standardized checklists.
- Simulation-based evaluation to demonstrate proficiency in managing simulated OASI cases before being certified for clinical practice.

##### Certification and Recertification

- Repair of third- and fourth-degree perineal tears is incorporated in the RCOG core training logbook and should be included in other national obstetric training curricula.
- Hospitals and professional organizations should implement certification programs to validate provider competency in OASI management.
- Recertification every 2–3 years ensures that providers stay up to date on evolving practices and guidelines. The effectiveness of training appears to decrease over time, hence the need for continuous or periodic training [319, 320].

##### Feedback Mechanisms

Continuous feedback is essential for improving provider performance. Supervisors should provide constructive feedback during training sessions, highlighting areas for improvement and acknowledging strengths.

#### Program Implementation

##### Institutional Support

Health care institutions must prioritize OASI training by allocating resources for educational programs and simulation facilities. Leadership should advocate for the integration of OASI education into routine obstetric training.

##### National and International Guidelines

National guidelines, such as those from the RCOG, emphasize the importance of provider education and competency in reducing OASI incidence [187]. International collaboration through organizations such as the IUGA can facilitate the sharing of best practices and training resources.

##### Monitoring and Evaluation of Training

The effectiveness of training programs should be evaluated through:

- Audit and feedback: Regular audits of OASI incidence rates and provider performance data.
- Patient outcomes: Monitoring long-term outcomes such as anal incontinence rates and patient satisfaction scores.

### Patient Education and Counselling

#### Antepartum Education

**The best time for this education and counseling is within the antenatal period (GPP).**

**Patients should be educated on pelvic floor anatomy and function and counseled about the potential for injury to the anal sphincter and/or levator ani that can result in symptoms of fecal incontinence, urinary incontinence, pelvic organ prolapse, and pain (GPP).**

**Engage patients in shared decision-making regarding modifiable risk factors for injury, such as episiotomy and OVB (GPP).**

**A consent discussion and documentation regarding episiotomy, OVB, and the potential for perineal laceration repair should be undertaken (GPP).**

#### Postpartum Counseling

**When an OASI occurs, patients should be informed of the nature of the tear, preferably using drawings and written information in their preferred language (GPP).**

**After sustaining an OASI, patients should be informed of the risks of pelvic floor dysfunction, especially anal incontinence symptoms (GPP).**

#### Reasons to Seek Care

**After sustaining an OASI, patients should be instructed to seek care immediately for any signs of dehiscence or infection, such as erythema, increased swelling or pain, skin-edge separation, malodorous discharge, or fever (GPP).**

A systematic review of 14 studies from multiple countries that included 3950 pregnant and postpartum participants found significant gaps in knowledge regarding pelvic floor anatomy and function, pelvic floor disorders, risk factors, and prevention measures [326]. Lower knowledge levels correlate with factors such as higher parity and age, low socioeconomic status, and low education level [327, 328]. In a study of 402 pregnant women, less than 50% showed knowledge proficiency regarding pelvic floor disorders [329]. Studies assessing knowledge specific to anorectal disorders show correct responses to only 35–40% of the questions [327, 330].

When reflecting on their pregnancy, postpartum women report that they wish that they had been provided with more information regarding the pelvic floor, self-care during recovery, and risks of injury with vaginal birth [69, 70]. Women who experience OASIs also report a desire for more postpartum information regarding their injury, recovery, and implications for their pelvic floor health [69, 70]. Health care providers are the primary source of information for patients, followed by sources such as written material, media/internet, and family/friends [330]. However, patient education from providers is often completely absent from antenatal and postpartum care, and when it is provided, it is often inadequate. In a study on pelvic floor knowledge in peripartum women, only 49% of participants received any information, and 75% reported that the information was inadequate [331]. Another survey study of 239 participants regarding their prenatal education revealed that 51% were not informed about episiotomy, 56% did not have information about forceps or vacuum, and 81% were not informed about the risk of fecal incontinence [71]. In women who sustained an OASI, one study revealed that only 68% of patients were told about their injury [75].

Education is essential for patients to make informed decisions regarding their health and is a key component of consent [66, 67]. Patients should be educated on pelvic floor anatomy and function and counseled about the potential for injury to the anal sphincter and/or levator ani that can result in symptoms of fecal incontinence, urinary incontinence, pelvic organ prolapse, and pain. They should be engaged in shared decision-making regarding modifiable risk factors for injury, such as episiotomy and OVB. The best time for this education and counseling is within the antenatal period, when a patient is not under the stress of labor and has the opportunity to consider the information, ask questions, and communicate their values [66, 67].

Educational interventions improve outcomes. In a study on education regarding perineal tears and episiotomy, patients reported that the information improved their understanding of childbirth and comfort with the birthing process. There was also a significant reduction in anxiety regarding episiotomy and perineal tears [77]. The concern that education will result in an increase in CS by maternal request is not evidence-based. A study of 40 primiparous pregnant women who attended a 2-h pelvic health workshop with counseling on the risks of CS, vaginal birth, and OVB did not change the participants’ preferences regarding mode of birth, and was well-received, with 98% reporting that they would recommend it to a friend [332].

When an OASI occurs, patients should be informed of the nature of the tear, preferably using drawings and written information in their preferred language [187]. They should be taught how to care for their wound and the importance of close follow-up. It is important to empower patients in their role of supporting the wound-healing process through self-care and to monitor their wounds for warning signs. They should be instructed to seek care immediately for any signs of dehiscence or infection, such as erythema, increased swelling or pain, skin-edge separation, malodorous discharge, or fever.

After sustaining an OASI, patients should be informed of the risks of pelvic floor dysfunction, especially anal incontinence symptoms. Research shows that patients do not necessarily seek care for pelvic floor disorders, owing to factors such as a lack of knowledge, over-normalization of symptoms as something that women just deal with, or embarrassment [16–18, 333–335]. It is therefore essential that health care providers directly ask about symptoms and educate patients on their increased risk of pelvic floor dysfunction, the importance of supporting adequate wound healing, the availability of therapies for incontinence, and the recommendation for close follow-up in a peripartum pelvic health clinic or with a provider experienced in the management of OASIs [187].

### Patient Information Leaflets, Links, Apps, Support Groups

**Patient education about perineal trauma, postpartum recovery, and the role of pelvic health physiotherapy is important for women both antepartum and postpartum (GPP).**

Health care providers need to educate women both antepartum and postpartum regarding perineal lacerations, postpartum pelvic floor recovery, and the role of pelvic health physiotherapy. Provision of psychological support for postpartum women with OASIs is also essential [160]. Support groups may be beneficial to provide ongoing emotional support and connect women with others who have experienced perineal trauma. Patient educational resources and support groups are presented in Appendix 1. Virtual options provide flexibility in accessing educational resources and connecting women globally [336] (Evidence level 4).

### Information Resources for Health Providers

Information regarding OASIs amongst health providers is limited. Lacking the knowledge, including the latest evidence, treatment guidelines, or best practices, may compromise the quality of care, leading to poorer patient outcomes. It is evident that knowledge and confidence in managing OASIs in most resource-limited settings are suboptimal, highlighting the need for ongoing training [337] and the availability of key information resources for health care providers on OASIs.

These resources will help to ensure that health care providers stay up to date on best practices for managing OASIs, with a focus on preventing complications and promoting recovery for affected individuals.

Although the guidelines above offer excellent direction, international guidelines can provide broader, more universally applicable recommendations that can help physicians in low-resource settings with limited local infrastructure and expertise. These guidelines can be crucial to ensure that even in resource-constrained settings, health care providers will have access to evidence-based protocols that promote the safety and well-being of both mothers and babies.

### Audits of Management and Outcome

Quality improvement is an important approach to risk management, such that improving obstetrical quality and safety should be a priority for providers, health authorities, and professional societies. Many quality-improvement interventions have been documented, including:

- Checklists.
- Safety bundles.
- Provider educational interventions.

There is evidence that approaches such as care bundles, skill-development training, and an awareness campaign improve obstetric outcomes, especially in SVBs [11]. However, gaps in perineal care management have been demonstrated even following successful implementation of quality-improvement programs [338]. This highlights the value of audit as a tool for sustaining quality improvement in the management of OASIs.

In addition to well-defined protocols for the prevention, identification, and management of OASIs, organizations should audit parameters to monitor compliance with these protocols. The first parameter to audit is the incidence of OASIs. The national incidence of OASIs varies significantly by jurisdiction, from 0.1 to 3.4% for SVB and 0.2 to 16.3% for OVB, reflecting different practice standards and the accuracy of measurement [339]. Consequently, the local incidence should be tracked against the national incidence. The variation in incidence by jurisdiction highlights the value of auditing documentation, especially when OASIs occur. The following should be audited:

- Type of anesthesia.
- Anatomical findings on systematic examination of the vagina, perineum, and rectum.
- Method of repair, including suture material.
- The level of specific training in OASIs of the clinicians involved in diagnosis and performing the repair.
- Information provided to the patient, including postoperative advice.
- Follow-up appointments.

Evidence from a jurisdiction without a national OASIs standard revealed significant variation in quality of care, with discrepancies attributed to power dimensions between providers and patients, and disagreement between professional groups regarding the scientific evidence for the suggested prevention and care of OASIs [340]. This provides a strong argument for developing national standards and an audit to assess compliance.

## Section 8: Concluding Sections

### Summary

Globally, OASI incidence has continued to rise over recent decades. OASIs are associated with significant short- and long-term sequelae, including anal incontinence, chronic perineal pain, sexual dysfunction, and psychological distress in up to half of patients. These tears are a leading cause of maternal morbidity, with serious long-term impacts on quality of life, which for some do not present until years after the initial OASI. The clinical repercussions for patients who experience OASI underscore the importance of thorough preventative, management, and educational strategies.

For providers, it is essential to have a thorough understanding of anorectal and perineal anatomy, as outlined in this document. Anal continence is a complex interaction between neuromuscular, hormonal, and cognitive factors. OASI results in both anatomical disruption and impairment of the complex neuromuscular physiology. As such, efforts aimed at primary prevention are vital; however, when these tears occur, ensuring accurate diagnosis, meticulous repair, and promotion of healing is paramount in limiting the associated clinical ramifications. A lack of provider knowledge and training is often cited as a contributing factor to diagnostic and therapeutic deficiencies in OASI care. Data support the efficacy of hands-on workshops and audiovisual materials to enhance OASI care and reduce underdiagnosis of this impactful condition.

For obstetric patients, early informed consent and antenatal discussion of pelvic anatomy, delivery risks, and interventions to mitigate these risks are essential. Although research highlights a variety of efficacious perineal protection, risk-reducing techniques, such as perineal massage, warm compresses, and selective MLE, these are not consistently implemented in maternal units. When an OASI does occur, it is important to obtain and document patient-informed consent for the ensuing careful examination and thorough repair, as well as to educate the patient on the laceration incurred and the necessary components of aftercare. When feasible, repair in an operating room by an experienced provider should be considered to improve the likelihood of the patient achieving a successful repair.

Close outpatient postpartum care with a specialist should be initiated following OASI, at which time examination, education, anticipatory guidance, and anorectal investigations to assess the structure and function of the anal sphincter complex can be performed. Patients should also be informed of the role of physiotherapy, which may be helpful to reduce a variety of sequelae. Measures aimed at secondary prevention for those who experience OASI are also important, as the risk of a repeat OASI, though low, still occurs in approximately 6%. This involves early third-trimester collaborative, patient-centered counseling to empower patient-informed decision-making. Published protocols to aid in mode-of-delivery counseling in a subsequent pregnancy exist and are a helpful tool for guiding this discussion. Further, these discussions and care plans should be thoroughly documented in the medical record.

There continue to be significant opportunities to enhance care provision prior to and at the time of primary delivery, as well as at the time of subsequent delivery for individuals who have a history of OASI. Future research will be essential to reduce risk and improve outcomes for this high-risk, unique patient population.

### Areas for Future Research

The constituents of the continence mechanism and how they interact to provide continence are well described. However, the significance, on ultrasonography, of a wide levator hiatus and potential contribution to the pathophysiology of incontinence and prolapse will require further investigation [37].

Increasingly, there is good evidence to support clinical algorithms for the treatment of OASI, although they utilize diagnostic technologies and treatments that may not be available in low-resource settings. Consequently, achieving parity for these centers will require research to support tiered implementation models.

Techniques for the initial repair of the EAS after OASI include the end-to-end and overlapping approaches. The best evidence to compare these two approaches is a Cochrane Review that showed no difference in flatal and fecal incontinence at 12 or 36 months. However, there was less fecal urgency and less deterioration of incontinence in the overlapping technique. Based on the limited number of studies and heterogeneity in outcomes, further research to compare these surgical techniques in terms of broader patient outcomes is warranted [90].

Although there is limited evidence that different maternal birth positions may impact risk factors for OASIs, including duration of the second stage of labor, use of episiotomy and OVB, and fetal malposition, there are inadequate data to link maternal birth position to the direct risk of OASIs [215, 218, 219].

Future research is required regarding the impact of the mode of subsequent birth on the development or progression of anorectal symptoms in women with a prior OASI. This includes high-quality research to determine the effect of the birth route on postpartum anorectal symptoms after subsequent birth post-OASI and in the long term to guide individualized decision-making [281]. Further research is also required to evaluate the effectiveness of intrapartum strategies in the prevention of rOASI during a subsequent birth, including the selective use of MLE, when clinically indicated, as a potentially effective intervention to reduce the risk of recurrence [282]. Finally, evidence of long-term regret and satisfaction related to the mode of subsequent birth post-OASI is limited [278].

## Conclusions

The development of this international guideline, which addresses identification, management, treatment of complications, long-term follow-up, and prevention of OASIs, addresses a critical gap in maternal health care. This guideline is distinct in both scope and inclusivity, through its strong clinical evidence base, international perspectives, and incorporation of the experiences of clinicians and health systems from low-resource contexts.

Current national and regional guidelines vary widely with regard to methodological quality and strength of evidence base. In addition, although they provide valuable direction, they are often designed for high-resource settings and lack applicability in health systems with limited infrastructure, workforce, and access to specialist services. As a result, many women globally remain without equitable access to evidence-based, effective and feasible care following OASI. By offering evidence-based but pragmatic, tiered recommendations that balance optimal evidence-based practice with resource-sensitive adaptations, this document provides a framework that is both scientifically rigorous and globally relevant.

## Appendix 1

### Resources for Women with Obstetric Anal Sphincter Injuries

The table below lists resources for women with OASIs.

**Table:**

Patient education	IUGA “Third and Fourth Degree Perineal Tears” leaflet https://www.yourpelvicfloor.org/conditions/third-and-fourth-degree-perineal-tears/
	RCOG “Perineal Tears and Episiotomies in Childbirth” https://www.rcog.org.uk/tears
	AUGS “Third and Fourth Degree Perineal Tears” https://www.voicesforpfd.org/assets/2/6/Perineal_Tears.pdf
	“Obstetrical Tears: A Patient Guide” https://obgynacademy.com/obstetrical-lacerations/
	Women’s Healthcare Australasia “Reducing Third and Fourth Degree Perineal Tears” https://women.wcha.asn.au/collaborate/breakthrough-collaboratives/perineal-tears/providing-information-and-engaging-women-in-decision-making
	Queensland Health Australia “Third and Fourth Degree Tears” https://www.health.qld.gov.au/__data/assets/pdf_file/0018/150363/c-peritears-34degree.pdf
	Australian Commission on Safety and Quality in Health Care “Information for Women—Third and Fourth Degree Perineal Tears Clinical Care Standard” https://www.safetyandquality.gov.au/standards/clinical-care-standards/third-and-fourth-degree-perineal-tears-clinical-care-standard/information-women-third-and-fourth-degree-perineal-tears-clinical-care-standard
	https://www.perineum.net
Emotional impact	Birth Trauma Association https://www.birthtraumaassociation.org
	Postpartum Support International https://www.postpartum.net
	PANDA https://www.panda.org.au
	PaNDAS PND Awareness and Support https://pandasfoundation.org.uk
	Make Birth Better https://pandasfoundation.org.uk
	Social, Psychological, and Emotional Impact of OASIs resources https://obgynacademy.com/obstetrical-lacerations/
	The Blue Dot Project https://www.thebluedotproject.org
	Birth Trauma Association “Fathers and Partners: Coping With a Traumatic Birth” https://static1.squarespace.com/static/645b78325e03ca278fdd150e/t/64d3b333a7d8fe0fd4e7761c/1691595578226/BTA_Fathers_and_Partners_-_Coping_with_a_Traumatic_Birth_PRINT_4pp_2_AW.pdf
Pelvic health physiotherapy	NHS Fife Postnatal Physiotherapy https://www.nhsfife.org/services/all-services/physiotherapy/pelvic-health-physiotherapy/postnatal-physiotherapy/
	OBGYN Academy “Role of Pelvic Health Physiotherapy Postpartum” https://www.youtube.com/watch?v=lqAfst9xX1E&t=2s
	Pelvic Obstetric and Gynaecological Physiotherapy “The Pelvic Floor Muscles—A Guide for Women” https://thepogp.co.uk/_userfiles/pages/files/resources/20818_pogp_pelvicfloor_for_women_signed_off_1.pdf
	Pelvic floor exercises postpartum https://www.mountsinai.on.ca/care/mount-sinai-perineal-clinic/pelvic-floor-exercises
Advocacy and support groups	Birth Trauma Association Peer Support https://www.birthtraumaassociation.org/peer-support
	MASIC Foundation (UK) https://masic.org.uk
	Australasian Birth Trauma Association (Australasia) https://birthtrauma.org.au/advocate/
	Postpartum Support International Birth Trauma Support Group https://postpartum.net/group/birth-trauma-support/
	SOLACE Foundation (USA) https://www.solaceforwomen.org

*AUGS* American Urogynecologic Society, *IUGA* International Urogynecological Association, *NHS* National Health Service, *OBGYN* obstetrics and gynecology, *PANDA* Perinatal Anxiety and Depression Australia, *PaNDAS* Pre- and Postnatal Depression Advice and Support, *PND* postnatal depression, *RCOG* Royal College of Obstetricians and Gynaecologists

## Appendix 2

### Components of Postpartum Assessment of Women with Obstetric Anal Sphincter Injuries

The table below show the history of women with OASIs.

**Table:**

Birth complicated by OASI	Birth date, duration since birth
	Birth details: location of birth, delivering provider, type of birth (spontaneous vs operative [type of instrument used, indication]), VBAC, gestational age at birth, birthweight, birth events, length of active and passive second stage, use of epidural, augmentation with oxytocin, presence of occiput posterior position, compound presentation, use and type of episiotomy, shoulder dystocia
	Degree of perineal laceration, presence and type of episiotomy
	Repair details: repair provider, repair technique, sutures used, rectovaginal examination (before and after the repair), analgesia, prophylactic antibiotics, laxatives
	Length of inpatient stay, intrapartum and postpartum complications
	Use of Sitz baths and Peri bottle, analgesia, laxatives
	Newborn feeding (breastfeeding, pumping, formula feeding), resumption of menses, use of birth estrogen
	Baby’s name, sex, and health
Short-term and long-term sequelae	• Timeline of symptoms: symptoms prior to all pregnancies, during each pregnancy, between pregnancies, postpartum after most recent birth, length of time for symptoms to improve/resolve, presence of residual symptoms
	Pad use, reason for pad use (to keep clean, urinary incontinence, lochia, menses, vaginal discharge, and/or fecal incontinence)
	Current and past treatments tried and response
	Anorectal symptoms:
	Bowel history: frequency of bowel movements, use of stool softener or constipating agent
	Fecal urgency, fecal incontinence (type of leakage (urge, passive, or combined), Bristol stool type, flatal incontinence, constipation, obstructive defecatory symptoms [341]
	Symptoms of rectovaginal fistula: foul-smelling vaginal discharge, passage of gas/pus/stool per vagina, recurrent urinary tract infections, recurrent vaginitis, dyspareunia [342]
	History of irritable bowel syndrome, inflammatory bowel disease
	Red-flag symptoms of colorectal cancer: melena or hematochezia, unexplained weight loss, anorexia, abdominal pain, abdominal distension, recent change in bowel habits (diarrhea or constipation), anemia, vomiting, weakness or malaise, family history of bowel cancer [343, 344]
	Urinary symptoms:
	Voiding history, urinary urgency, frequency, nocturia
	Urinary incontinence (type, amount, frequency)
	Fluid intake, use of bladder irritants, abdominal stressors
	Signs of wound infection/dehiscence (fever >38°C, chills, worsening perineal pain, severe abdominal pain, abnormal vaginal discharge, erythema or swelling around the wound, wound breakdown, malaise)
	Pelvic organ prolapse: bulge symptoms
	Sexual function: resumption of sexual activity since birth (fears or concerns if no resumption of sexual activity), urinary and/or anorectal symptoms during intercourse, decreased sexual desire, decreased arousal, anorgasmia or difficulty in achieving orgasm, reduced vulvovaginal sensation
	Pain: presence of pain (dyspareunia, dysuria, dyschezia, perineal pain, pubic pain, coccygeal pain)
	Psychological impact: history of mental health conditions prior to and during pregnancy, postpartum mental health screening (anxiety, depression, PTSD)
Impact on quality of life	Impact of OASI and its associated sequelae on daily activities, sexual activity, relationship with partner and/or family, emotional well-being, and body image
	Difficulty coping, social support
Pelvic floor assessment and treatment	Pelvic health physiotherapy assessment
	Pelvic floor muscle training or pelvic floor muscle relaxation (if hypertonic pelvic floor)
Family planning	Future fertility planning: desire and timing of subsequent pregnancies
	Contraception use
	Patient’s preferred mode of subsequent birth, concerns regarding birth
	Obstetrical history
	Gynecological history
	Past medical history: medical conditions, medications, allergies, blood transfusions
	Surgical history
	Family history
	Social history
Concerns	Patients and families’ concerns and expectations

*OASIs* obstetric anal sphincter injuries, *PTSD* post-traumatic stress disorder, *VBAC* vaginal birth after cesarean section

The table below lists questionnaires that can be utilized for women with OASIs.

**Table:**

Anorectal symptoms	St. Mark’s Incontinence Score [287]
Pelvic floor dysfunction	Pelvic Floor Distress Inventory [345]
Sexual function and perineal pain	Female Sexual Function Index [346]
	Visual Analog Pain Scale [347]
	Leeds Assessment of Neuropathic Symptoms and Signs Pain Scale for neuropathic pain (Bennett 2001) [348]
	Personal Assessment of Intimacy in Relationships [349]
Quality of life	Fecal incontinence quality of life scale [173]
	Pelvic Floor Impact Questionnaire-short form 7 [350]
Mental health	Edinburgh Postnatal Depression Scale [351]
	Postpartum Specific Anxiety Scale Short-Form [352]
	City Birth Trauma Scale [353]
	Female Genital Self-Image Scale [354]
Patient satisfaction	Patient Satisfaction Score (five-point Likert scale) (there is currently no validated patient satisfaction scale in women with OASIs)

*OASIs* obstetric anal sphincter injuries

The table below shows information on the physical examination of women with OASIs.

**Table:**

General appearance, BMI, vital signs	
Abdominal examination	Scars, lesions, areas of tenderness (allodynia/hyperesthesia, trigger points, Carnett’s sign), masses, hernias, diastasis recti, palpation of the pubic symphysis for pain
Inspection of perineum	Health of the urogenital mucosa (hypoestrogenism, vulvovaginitis, vulvar dermatosis, labial fusion, excoriation, hematoma), abnormal vaginal discharge, vaginal bleeding, presence of undissolved suture, granulation tissue, lesions, masses, band at posterior fourchette
	Perineum: perineal body length, deficient perineum,^a^ traumatic cloacal defect, evidence of episiotomy, perineal scar, wound infection/dehiscence, perineal abscess, skin tag, hemorrhoids, anal fissures, dovetail sign^b^
	Cough test: urethral hypermobility, presence of stress urinary incontinence
Neurological examination	Sensory examination using Q tip: loss of sensation, allodynia, hyperalgesia (ilioinguinal, genitofemoral, pudendal nerves)
	Cotton swab test (Q tip) of vestibule for vulvodynia^c^
	Sacral reflexes: bulbocavernosus and anocutaneous reflexes^d^
Speculum examination	Hematoma, presence of fecal matter in the vagina, vesicovaginal and rectovaginal fistula
	Examination for pelvic organ prolapse
Single-digit vaginal examination, bimanual examination	Presence of vaginal and/or introital narrowing, scar tissue
	Assessment for presence of levator avulsion^e^
	Musculoskeletal assessment for tone and pain^f^: superficial perineal muscles (ischiocavernosus, bulbospongiosus, perineal body, superficial transverse perineal muscle), levator ani (puborectalis, pubococcygeus, iliococcygeus), coccygeus, obturator internus, piriformis
	Modified Oxford Scale for pelvic floor muscle strength [355]
	Bimanual examination: uterine size and mobility, pelvic masses, tenderness
Rectovaginal examination, DRE	Assessment for rectovaginal fistula ± methylene blue test^g^
	Anal resting tone and anal squeeze contraction using DRESS score^h^ [307]
	Sustained anal sphincter squeeze over 5–10 s
	Involuntary contraction of anal sphincter complex on cough or Valsalva

*BMI* body mass index, *DRE* digital rectal examination, *DRESS* Digital Rectal Examination Scoring System, *OASIs* obstetric anal sphincter injuries

^a^Perineal body length <2.5 cm) [356]

^b^Absence of anterior perianal folds during contraction of the external anal sphincter suggestive of an external anal sphincter defect [357]

^c^During cotton swab test (Q-tip test), the vestibule is tested for pain at 1, 4, 6, 7, and 11 o’clock positions [358]

^d^Bulbospongiosus reflex is elicited by applying pressure on the clitoris or touching the labia minora lateral to the clitoris. To elicit the anocutaneous reflex, a pinprick is applied to the mucocutaneous junction of the anus. Contraction of the anal sphincter with these reflexes indicates an intact spinal reflex arc [359]

^e^Palpation is performed for detachment of the puborectalis muscle from its insertion on the inferior pubic ramus [360]

^f^Meister scale can be utilized for screening for pelvic floor myofascial pain [361]. Reissing scale can be utilized for pelvic floor muscle tone [362]

^g^Instill 50 ml of methylene blue using a Foley catheter into the rectum while inspecting the posterior wall of the vagina using a speculum for leakage of blue dye into the vagina, which is indicative of a rectovaginal fistula [363]

^h^Resting tone reflects internal anal sphincter function, and the squeeze contraction reflects external anal sphincter function [46]

^i^Normal endurance squeeze pressure ≥5 s [3]
